# Hypoxia-driven mobilization of altruistic cancer stem cells in platinum-treated head and neck cancer

**DOI:** 10.3389/fimmu.2024.1336882

**Published:** 2025-02-03

**Authors:** Lekhika Pathak, Bidisha Pal, Joyeeta Talukdar, Partha Jyoti Saikia, Sorra Sandhya, Wale Tasabehji, Hong Li, Jyotirmoy Phukan, Anjan Bhuyan, Sanjukta Patra, Bikul Das

**Affiliations:** ^1^ Department of Cancer and Stem Cell Biology, KaviKrishna Laboratory, Indian Institute of Technology Guwahati Research Park, Guwahati, Assam, India; ^2^ Department of Medical Oncology, KaviKrishna Telemedicine Care, Sualkuchi, Assam, India; ^3^ School of Agro and Rural Technology, Indian Institute of Technology, Guwahati, Assam, India; ^4^ Department of Experimental Therapeutics, Thoreau Laboratory for Global Health, University of Massachusetts, Lowell, MA, United States; ^5^ Department of Immunology and Infectious Diseases, Forsyth Institute, Cambridge, MA, United States; ^6^ Department of Otorhinolaryngology, Guwahati Medical College, Guwahati, Assam, India

**Keywords:** circulating tumor cells, head and neck squamous cell carcinoma (HNSCC), tumor stemness defense (TSD), cancer stem cells (CSCs), tumor hypoxia, platinum chemotherapy, altruistic stem cells (ASCs), reawakening CSCs (R-CSCs)

## Abstract

**Background:**

Head and neck cancers harbor dormant cancer stem cells (CSCs). This study explores how platinum therapy impacts these cells in a non-genetic manner and the role of hypoxia in this process. Previously, we identified a novel population of CSCs exhibiting an “altruistic” phenotype, sacrificing self-renewal to promote niche defense (tumor stemness defense, TSD), potentially protecting a dormant subpopulation of CSCs, the reawakening CSC (R-CSC) retaining stress memory. This TSD phenotype involves the activation of the MYC-HIF2α pathway and, importantly, is linked to a hypoxic tumor microenvironment. We termed these TSD+ CSCs “altruistic cancer stem cells” (A-CSCs). Here we investigated the potential role of tumor hypoxia in the mobilization of TSD+ CSCs to the circulation as a part of niche defense against platinum therapy.

**Methods:**

We isolated CTCs and primary tumor cells from head and neck squamous cell carcinoma (HNSCC) patients undergoing platinum therapy (*n* = 14). We analyzed the TSD phenotype and markers of hypoxia in these cells. Additionally, we further characterized a previously reported pre-clinical model of platinum-induced tumor stemness to study the link between hypoxia, TSD+ CSC emergence, and mobilization to the circulation and bone marrow.

**Results:**

We isolated TSD+ CTCs with a hypoxic signature from eight out of 14 HNSCC patients. These cells displayed increased proliferation and invasion upon cisplatin treatment, suggesting a role in niche defense. Our pre-clinical model confirmed that hypoxia directly correlates with the expansion of TSD+ CSCs and their mobilization into the circulation and bone marrow following cisplatin treatment. We demonstrated the protection of R-CSCs by TSD+ CSCs. Notably, inhibiting hypoxia alone with tirapazamine did not reduce TSD+ CSCs, CTCs, or R-CSCs. However, combining tirapazamine with FM19G11, a MYC-HIF2α pathway inhibitor, significantly reduced the platinum-induced expansion of both TSD+ CSCs, CTCs, and the presence of R-CSCs in the bone marrow.

**Conclusions:**

This study reveals that HNSCC patients undergoing platinum therapy can harbor TSD+ CTCs exhibiting an altruistic phenotype and a hypoxic signature. Additionally, the pre-clinical study provides a novel non-genetic mechanism of therapy resistance-the altruistic tumor self-defense. The tumor microenvironment, through the emergence of TSD+ CSCs, appears to act collectively to defend the tumor self-identity by hijacking an altruistic stem cell niche defense mechanism.

## Introduction

Head and neck squamous cell carcinoma (HNSCC) remains a prevalent cancer with poor outcomes in India, often diagnosed at late stages and associated with low survival rates. Despite advancements in diagnosis and treatment, the incidence of HNSCC is projected to rise by 30% by 2030 (1). Notably, India bears a significant burden, with the highest global burden of oral cavity cancer (1). Cisplatin-based chemoradiation is the standard treatment for locally advanced HNSCC, including oral cancer (2–8). However, despite treatment, approximately 50% of stage III or IV cases experience locoregional or distant relapse, and 35% require adjustments due to cisplatin-induced toxicity (8). This underscores the need for novel therapeutic strategies, particularly those that target the mechanisms of therapy resistance.

Our research focuses on the role of the tumor microenvironment, specifically the cancer stem cell (CSC) niche defense, in promoting tumor self-defense and therapy resistance. We previously demonstrated that cisplatin treatment can induce an aggressive phenotype in the dormant migratory side-population (SPm) fraction of various solid tumor cell lines, the tumor stemness switch phenotype (9). The SP fraction has been characterized in HNSCC (10, 11). Subsequently, we showed that this tumor stemness switch or the tumor stemness defense (TSD) phenotype can be isolated from the HNSCC cell lines exposed to extreme hypoxia and oxidative stress. The EpCAM+ABCG2+ CSCs were enriched in the TSD phenotype (12). We speculate that the TSD phenotype might represent the reprogrammed state of CSCs activated by the niche defense mechanism against external stress.

CSCs reside within a specialized microenvironment called the niche, which plays a crucial role in supporting their self-renewal, differentiation, and therapy resistance. In healthy tissues, stem cells can activate a “niche defense mechanism” when the niche is stressed. This involves transient reprogramming where they lose the tumor suppressor p53 and activate specific signaling pathways, sacrificing their long-term fitness to protect the niche and ensure the survival of the stem cell population. This strategy, termed “altruistic stemness defense,” highlights a surprising level of cooperation within the stem cell compartment to preserve self-identity (13, 14).

We hypothesize that a similar phenomenon might occur in HNSCC. Platinum-induced stress may activate the CSC niche defense mechanism, reprogramming CSCs within the niche to the TSD phenotype. These TSD+ CSCs, which we propose are best characterized as “altruistic cancer stem cells (A-CSCs)”, may then detach from the tumor and enter circulation as circulating tumor cells (CTCs). Identifying these circulating A-CSCs could offer a valuable window to monitor treatment response and potentially predict a relapse risk. The rationale lies in our hypothesis that the TSD phenotype serves a protective role, safeguarding the CSC niche and also securing metastatic niches for the future re-seeding of the tumor (12, 15).

The presence of CTCs in the bloodstream is associated with poor prognosis and an increased risk of metastasis in various cancers (16). In HNSCC, EpCAM+ and EpCAM- CTCs were identified and found to be associated with poor prognosis (16, 17). However, the biology of these CTCs remains poorly understood. Studying CTCs exhibiting the TSD phenotype offers a unique opportunity to understand the activation of the CSC niche defense in HNSCC. By identifying and characterizing these cells, we can investigate their potential as a biomarker for this process. If circulating A-CSCs are indeed a consequence of CSC niche defense activation, their presence could indicate a heightened risk of therapy resistance and relapse-2 key aspects of tumor self-defense (14).

However, isolation and characterization of CTCs are challenging, as these cells are very rare and difficult to expand in *in vitro* cell culture media. In this context, we have developed an *in vitro* culture media that can sustain the stemness of the TSD phenotype. The special conditioned media, the “injured conditioned media (ICM)”, is obtained by exposing the mesenchymal stem cells (MSCs) to oxidative stress and hypoxia for 4 days and then collecting the conditioned media (15). To elicit the altruistic behavior of TSD+ CSCs, we have characterized an *in vitro* culture method of serum deprivation and 2% oxygen to mimic the hypoxic tumor microenvironment (TME).

In this study, we aimed to identify CTC exhibiting the TSD phenotype in HNSCC patients undergoing cisplatin-based chemoradiation. We further examined whether the primary tumors of patients with the CTCs exhibit the tumor-derived CSCs with TSD phenotype. Next, we wanted to characterize the altruistic behavior of the CTCs with TSD phenotype. Additionally, we sought to develop a pre-clinical model to elucidate the potential role of CTC with TSD phenotype (i.e., C-ASCs) as a biomarker for the activation of the CSC niche defense in HNSCC.

We successfully isolated EpCAM+/ABCG2+ cells from the circulation of HNSCC subjects. These sorted cells were then evaluated for their TSD phenotype using a comprehensive set of assays, including studies on transient growth expansion in ICM, self-sufficiency, expression of TSD phenotype genes (including the autocrine growth receptors and factors involved in MYC-HIF-2alpha stemness pathway), and their altruistic behavior using the assay we established for altruistic stem cells (ASCs). The pre-clinical model showed that TSD+ CSCs protect a dormant CSC population with the ability to retain stress memory and potentially reawaken upon future therapeutic challenges, the R-CSCs. Therefore, the ability to identify both A-CSCs and R-CSCs using a pre-clinical model now provides significant advantages to study the altruistic CSC phenotype and its role in tumor progression, therapeutic resistance, and metastasis.

## Methodology

### Isolation of circulating and primary EpCAM+/ABCG2+ cells

**Figure 1:**
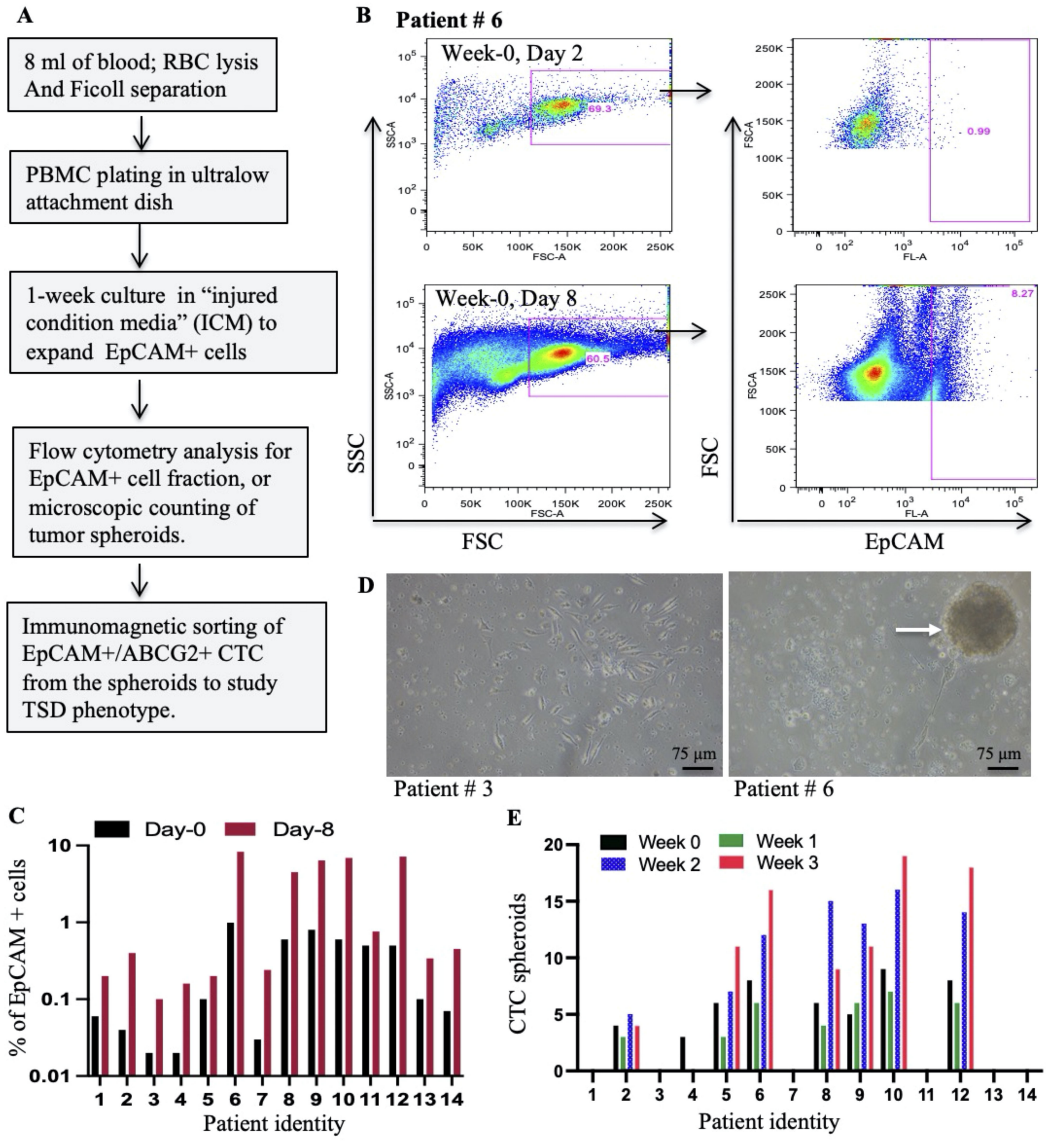
Isolation and expansion of circulating EpCAM+ cells from HNSCC patients. **(A)** Schematic workflow depicts the process of isolating and expanding EpCAM+ spheroids from PBMCs collected during cisplatin chemotherapy. **(B)** Representative flow cytometry plots illustrate the strategy for identifying EpCAM+ cells within PBMCs at baseline (Day 2) and after culture with a special media; injured conditioned media (ICM) (15). **(C)** Flow cytometry analysis was used to quantify the percentage of EpCAM+ cells within PBMCs following *in vitro* culture with ICM. **(D)** Phase-contrast microscopy images show PBMC cultures at Day 8 (Week 0): left image (patient #3) shows individual cells, while the right image (patient #6) highlights the formation of multicellular spheroids (arrows). **(E)** The number of spheroids formed from PBMCs grown in ICM for 8 days is quantified by manual counting. Week 0 corresponds to the day of chemotherapy administration, while Weeks 1-3 represent subsequent weeks.

The clinical study was approved by the Institutional Ethics Committee of KaviKrishna Laboratory and Guwahati Medical College and Hospital (Guwahati, Assam, India) as per the Declaration of Helsinki. We selected 14 out of 32 HNSCC subjects (**Supplementary Table S1**) being monitored in our clinic during the last 15 years through a community-based participatory action research (CBPAR) initiative (18, 19). The 14 subjects (male/female, 35–65 years old) were given cisplatin monotherapy either as neoadjuvant, adjuvant, or second-line therapy after relapse. Patient information, including demographics and treatment details, is provided in a supplementary table (**Supplementary Table S1**). Subjects with pregnancy, diabetes, heart diseases, low hemoglobin (<8 mg/dl), and low WBC count (<3,000 cells/µL blood) were excluded from the study. Patient information was obtained from the histopathology report, CT scan report, as well as treatment details card and certified by an in-house pathologist at KaviKrishna Telemedicine Care (KTC). For the CTC study, three blood samples were collected from each patient during the second cycle of the cisplatin-based chemotherapy, starting from day 0 (**Figure 1A**). After obtaining written informed consent, we have collected primary tumor samples from the patients (2, 4, 6, 10, 12, 14) being selected for surgery with or without neoadjuvant chemotherapy or radiation. Before surgery (48 h before), the patients were given oral pimonidazole (500 mg/m^2^ orally 20–24 h before surgery). The primary tumors were dissociated and then stored in liquid nitrogen at KaviKrishna Lab’s Institutional Biobank. Tumor tissues were dissociated and subjected to flow cytometry analysis and immunomagnetic sorting using the KKL protocol as described (20). Briefly, primary tumor tissue (~1 g of tissues) was collected post-surgery and transported in Hanks balanced salt solution (HBSS; Sigma-Aldrich) and 2.5% human albumin to the KaviKrishna Lab. The tissue was dissociated using dispase (1 h at 37°C incubation), subjected to RBC lysis, and then washed twice in HBSS/human albumin solution. The dissociated tumor cells were mixed with DMSO and stored in liquid nitrogen for future use.

For the CTC collection, 5–10 mL of blood was collected from selected subjects (*n* = 14) for 3 weeks (once/week), starting from 21 days after the first cycle of cisplatin chemotherapy, that is day 0 of the 2nd cycle (week 0). The blood collection process was repeated once weekly for the next 3 weeks (week 1, week 2, and week 3). The collected blood was subjected to RBC lysis followed by Ficoll separation (21), plated in an ultra-low attachment dish, and then *in vitro* cultured in the “injured conditioned media” (ICM) (15). The cells were subjected to flow cytometry analysis for EpCAM+ cells and immunomagnetic sorting of EpCAM+/ABCG2+ cells after 2 to 8 days of *in vitro* culture. Briefly, bone marrow mesenchymal stem cells (BM-MSCs) obtained from healthy volunteers and expanded in serum-rich DMEM/F12 media (10% horse serum, 10% FBS, 50 uM 2-mercaptoethanol, and 1 uM hydrocortisone) were exposed to extreme hypoxia (<0.1% oxygen) followed by re-oxygenation plus hydrogen peroxide for 3 days. The media was half-changed with fresh media (serum-free media DMEM/F12 media enriched with HEPES, glutamine, sodium pyruvate, 400 ng/mL hydrocortisone, and 50 uM 2-mercaptoethanol), and the cells were allowed to grow for 1 week. The total media (5 mL/10^6^ MSCs) were collected, and SDF-1alpha was measured. The media batch having SDF-1 alpha concentration of <1.5 ng/10^6^ cells/mL was stored as ICM for future use. The PBMC grown in the ICM facilitated the formation of tumor spheroids, and these spheroids were dissociated and subjected to immunomagnetic sorting for EpCAM+ cells (Antibody #ab213500, Abcam, conjugated with fluorescein isothiocyanate (FITC) by using a SiteClick antibody labeling kit). The EpCAM+ cells were then expanded for 2 to 3 days in the ICM and subjected to immunomagnetic sorting for ABCG2+ cells by our established protocol as described (20, 22). Briefly, the ABCG2 antibody (#ab3380, Abcam) was conjugated with PE by using a SiteClick antibody labeling kit and then utilized for immunomagnetic sorting of ABCG2+ cells. These EpCAM+/ABCG2+ cells were then either further expanded for 7 days ICM or grown in serum-free DMEM/F12 media (enriched with HEPES, glutamine, sodium pyruvate, 400 ng/mL hydrocortisone, and 50 uM 2-mercaptoethanol) to study the altruistic behavior as previously described (12, 23). When required, the cells were cultured in 2% oxygen by using a hypoxia-incubation chamber flushed with pre-mixed gas (2% oxygen, 10% CO_2_, and 88% N_2_) as previously described (20). In selected cases, PBMC grown in ICM for 2 to 3 days were directly immunomagnetically sorted EpCAM+/ABCG2+ cells, and the sorted cells were cultured in collagen-coated chamber slides for 4–6 h to perform immunofluorescence (IF).

### *In vitro* cell culture to study platinum-induced TSD+ phenotype in HNSCC

All of the experimental protocols were approved and undertaken following the guidelines of the Institutional Ethics Committee, Institutional Bioethics Committee, KaviKrishna Laboratory (Guwahati, Assam, India), Forsyth Institute (Cambridge, MA, USA) and the University of Massachusetts, Lowell, MA, USA. The SCC 25 cell line was obtained from ATCC, and cells were cultured in Dulbecco’s modified Eagle’s medium containing Ham’s F12 (DMEM F-12) in the ratio of 1:1. DMEM F-12 is enriched with sodium bicarbonate (1.2 g/L), 2.5 mM L-glutamine, 15 mM (4-(2-hydroxyethyl)-1-piperazineethanesulfonic acid (HEPES), and 0.5 mM sodium pyruvate (catalog no. 11330-057, Gibco). The medium was supplemented with hydrocortisone (400 ng/mL; catalog no. H0888, Sigma) and 10% fetal bovine serum (catalog no. 16000-044) and used as complete isolation media. The cells were maintained as previously described in a humidified atmosphere of 5% CO_2_ at 37°C (15, 20). At least 1 M SCC-25 cells were sorted to obtain EpCAM+/ABCG2+ CSCs using an immunomagnetic sorter. The sorted cells were treated with cisplatin (3–10 μm) and cultured in serum-free DMEM/F12 media, supplemented with hydrocortisone (400 ng/mL), HEPES, glutamine, sodium pyruvate, and 50 uM 2-mercaptoethanol for 3 to 4 days. The cells were used to isolate migratory side-population cells or EpCAM+/ABCG2+ cells.

### Isolation of migratory and non-migratory side population cells

The side population (SP) cells from cisplatin-treated and untreated SCC-25 cells were obtained using the Hoechst 33342 dye (4.5 μg/mL/10^6^ cells) exclusion method as previously described (15). The Hoechst-treated cells were incubated at 37°C for 45 min and finally counterstained with 1 μg/mL propidium iodide (PI) to label dead cells and cellular debris. The PI-stained SP cells were subjected to flow cytometry to sort and analyze the SP population as explained before (15). A Boyden chamber invasion assay was performed to isolate the SPm and SPn populations from the sorted SP population as previously described (15). Briefly, polyvinyl membrane-based chambers (Corning Life Sciences, Lowell, MA, USA) were coated with ice-cold Matrigel (BD Biosciences, San Diego, CA, USA) and incubated at 37°C for 4 h. The sorted SP cells were added to the upper chamber, whereas the lower chamber was filled with DMEM/F12 media and incubated at 37°C for 8–24 h. The migratory cells were counted as SPm cells using crystal violet staining. The non-invading cells were also isolated and counted as SPn cells.

### Immunomagnetic sorting, flow cytometry analysis, and culture of EpCAM+/ABCG2+ population for TSD phenotype

The SCC-25-derived migratory SP (SPm) population was subjected to immunomagnetic sorting EpCAM+ cells and then ABCG2+ cells as described above. We have been using this method with a confirmed purity of 92–94% EpCAM+/ABCG2+ population (12).

### Real-time quantitative RT-PCR

Real-time quantitative PCR was performed as previously described (12) using TaqMan primers at 40 cycles with 100 ng of starting cDNA. GAPDH was used as an endogenous control. The RNA was quantified by the delta-delta CT method using Q-Rex software version 1.1 (Rotor-Gene Q-Qiagen, New Delhi, India). The following TaqMan gene expression primers were used: Human: BMI (Hs00995536_m1), ALDH1 (Hs00180254_m1), VEGFR1 (Hs01052961_m1), CXCR4 (Hs00976734_m1), TLR2(Hs02621280_s1), TLR4 (Hs00152939_m1), EGFR (Hs01076090_m1), EpCAM (Hs00901885_m1), PDL1 (Hs00204257_m1), GCL (Hs01031542_m1), p21 (Hs00355782_m1), MKI67 (Hs04981441_m1), CD47 (Hs00179953_m1), vimentin (Hs00958111_m1), snail (Hs00195591_m1), twist (Hs01379963_m1), Taz (Hs00210007_m1), ODCI (Hs00229049_m1). HIF -1a (Hs00153153_m1), HIF -2a (Hs01026149_m1), Oct 4 (Hs03005111_g1), Sox2 (Hs00602736_s1), CD44 (Hs01075862_m1) MYC (Hs00153408_m1), GAPDH (Hs00266705_g1), Nanog (Hs02387400_g1).

### Knockdown or inhibition of genes or pathways

Inhibition of human HIF-2α was achieved by using Accell siRNA as previously described (12, 20). Briefly, 1 μM Accell siRNA diluted in Accell siRNA delivery media was added to ABCG2+ cells. Gene silencing was confirmed by real-time PCR and western blot/ELISA. The *in vivo* inhibition of HIF-2α was achieved by treating mice with FM19G11(Millipore, Billerica, MA, USA) dissolved in 1.5% DMSO as follows: 5 mg/kg, i.p., thrice weekly for 2 weeks, or vehicle, 1.5% DMSO containing saline (100–500 μL volume for each injection for mice weighing 20 g). Terazapamine, 50 mg/kg, i.p., was added in selected groups. For the inhibition of hypoxia as well as VEGF/VEGFR1 and EGF/EGFR1, the mice were treated with axitinib (25 mg/kg daily for 2 weeks, i.p.) and erlotinib (80 mg/kg daily for 2 weeks, i.p.), respectively.

### *In vivo* tumorigenic assay

All of the experimental protocols were approved and undertaken in accordance with the guidelines of Forsyth Institute (Cambridge, MA, USA) and KaviKrishna Laboratory (Guwahati, Assam, India) institutional animal ethics committee. NOD/SCID mice were maintained in an AC animal facility before the experiments. Briefly, viable human primary tumors or SCC-25 cell line-derived EpcAM+/ABCG2+ and other cell types mentioned in the text were mixed with Matrigel (50 µL of cell suspension in 50 µL of Matrigel) and injected subcutaneously into both flanks of female nude mice (NOD/SCID). For the additional cisplatin treatment, after a week, the mice were injected intraperitoneally with cisplatin, 10 mg/kg, once weekly for 2 weeks (24) to induce cisplatin-mediated tumor stemness (9). Tumor size was measured as previously described (12, 15). The tumors were dissociated into cell suspensions in stipulated periods for EpCAM+/ABCG2+ cells. The dissociated tissues are also subjected to H&E stain for the tissue morphology study. Serial transplantation assay was performed as previously done (20). The CSC frequency was obtained using an extreme dilution limiting assay (ELDA).

### Detection of tumor hypoxia in HNSCC subjects and mice

To the selected human subjects, pimonidazole was given orally (500 mg/m^2^ orally 20–24 h before blood collection). The blood-derived ABCG2+/EpCAM+ cells were stained with human-specific pimonidazole antibody and subjected to FACS or fluorescence microscope. The human tumor cells were subjected to flow cytometry analysis for pimonidazole as previously described (15). For the xenograft study of tumor hypoxia, we followed our previously described lab protocol (15). Briefly, pimonidazole hydrochloride (N1165, Sigma Aldrich) was injected (60 mg/kg, i.v.) in tumor-bearing mice. After 2 h, tumor tissue was obtained and fixed in 10% paraformaldehyde. Sections of hypoxic tumor cells were stained with a monoclonal pimonidazole antibody (Chemicon; Millipore) for quantification by fluorescence-activated cell sorting (FACS) or detection by using a fluorescence microscope.

### Immunofluorescence and immunohistochemistry

The patients’ CTC-derived EpCAM+/ABCG2+ cells and SCC-25-derived EpCAM+/ABCG2+ cells were fixed with 4% paraformaldehyde for 10–20 min on a coverslip as previously described (12). Overnight incubation was done with primary antibody followed by secondary antibody as per the manufacturer’s protocol. Xenograft-derived tumor tissue was dissociated into a cell suspension and stained for EpCAM as well as ABCG2. Images were obtained using confocal microscopy.

### ELISA

The conditioned media of day 8 culture from different groups was collected and subjected to ELISA to measure VEGF, PIGF, SDF-1alpha, and HMGB1 (R&D ELISA kit) according to the manufacturer’s protocols. The ELISA for HIF-2alpha, MYC, Nanog, Sox2, and Oct-4 was performed as previously described (13, 20). The absorbance was measured at 450 nm using the iMarkMicroplate Absorbance Reader (Bio-Rad, Gurgaon, India).

### Western blotting

Western blot (WB) analysis was done as previously described (20) by using a 4%–12% sodium dodecyl sulfate-polyacrylamide gel and polyvinylidene difluoride membranes (Immobiolon-P, MilliporeSigma, cat. # IPVH20200), as previously described (15, 20). 50μg of the clear protein lysate was used. We have used the following antibodies: beta-actin (# BB-AB0024S, Biobharti, Kolkata, India), Myc (# BB-AB0045S, Biobharti, Kolkata, India), EpCAM (Abclonal, USA) and ABCG2 (Cell Signaling Technology, USA).

### Measurement of GSH and squalene

The conditioned media of TSD+ EpCAM+/ABCG2+ cells were subjected to GSH and squalene as previously described (24).

### Statistical analysis

The data are presented as mean ± standard error of the mean (SEM). The statistical calculations were performed with Graph-Pad Prism 10.0 (Hearne Scientific Software, Chicago, IL, USA) by using Student’s *t*-test for cell survival assays. ELISA and qPCR data were analyzed using one-way ANOVA with Tukey’s HSD test. Furthermore, analysis for tumorigenic potential was done by extreme limiting dilution assay (ELDA) (25). Statistical significance was set at *P <*0.05.

## Results

### Isolation of EpCAM+ circulating tumor cells from platinum-treated HNSCC patients

This study aimed to investigate the presence of EpCAM+ CTCs exhibiting the TSD phenotype in the peripheral blood of HNSCC patients treated with cisplatin. Blood samples collected from study subjects (*n* = 14; **Supplementary Table S1**) were processed using RBC lysis followed by Ficoll-based density gradient separation to isolate peripheral blood mononuclear cells (PBMCs) as well as tumor cells. The isolated cells were subjected to flow cytometry analysis for EpCAM+ cells following a week of growth in a special conditioned medium, the “injured conditioned media (ICM)”, that favors the growth of CSCs with TSD phenotype (15) (**Figure 1A**). We confirmed that ICM does not induce, but expand the TSD+ phenotype (**Supplementary Figure S1A, B**). The CTC present in the PBMC continued to grow in the ICM for a week to form multicellular tumor spheroids. The number of spheroids formed was used as an indicator of the presence of TSD phenotype in the CTCs. Results are given in the **Figure 1**. First, flow cytometry analysis of the PBMCs revealed the presence of 0.1-1.1% EpCAM+ cells in all patient samples on day 2 of *in vitro* culture, throughout the second chemotherapy cycle (weeks 0-3; **Figure 1B**, **Supplementary Figure S1C**). Second, these EpCAM+ cells underwent significant expansion (2-20-fold) during spheroid culture (**Figure 1C**), demonstrating the effectiveness of the ICM in expanding the CTCs. However, only 7 out of 14 patients (patients #2, 5-6, 8-10, and 12) showed significant spheroid formation (2-16 spheroids per 1x10^5 PBMCs) of CTC obtained during weeks 0-3 of chemotherapy (**Figures 1D, E**). This study provides preliminary evidence for the presence of EpCAM+ CTCs with a potential TSD phenotype in cisplatin-treated HNSCC patients.

### The EpCAM+ CTC exhibits TSD phenotype

**Figure 2:**
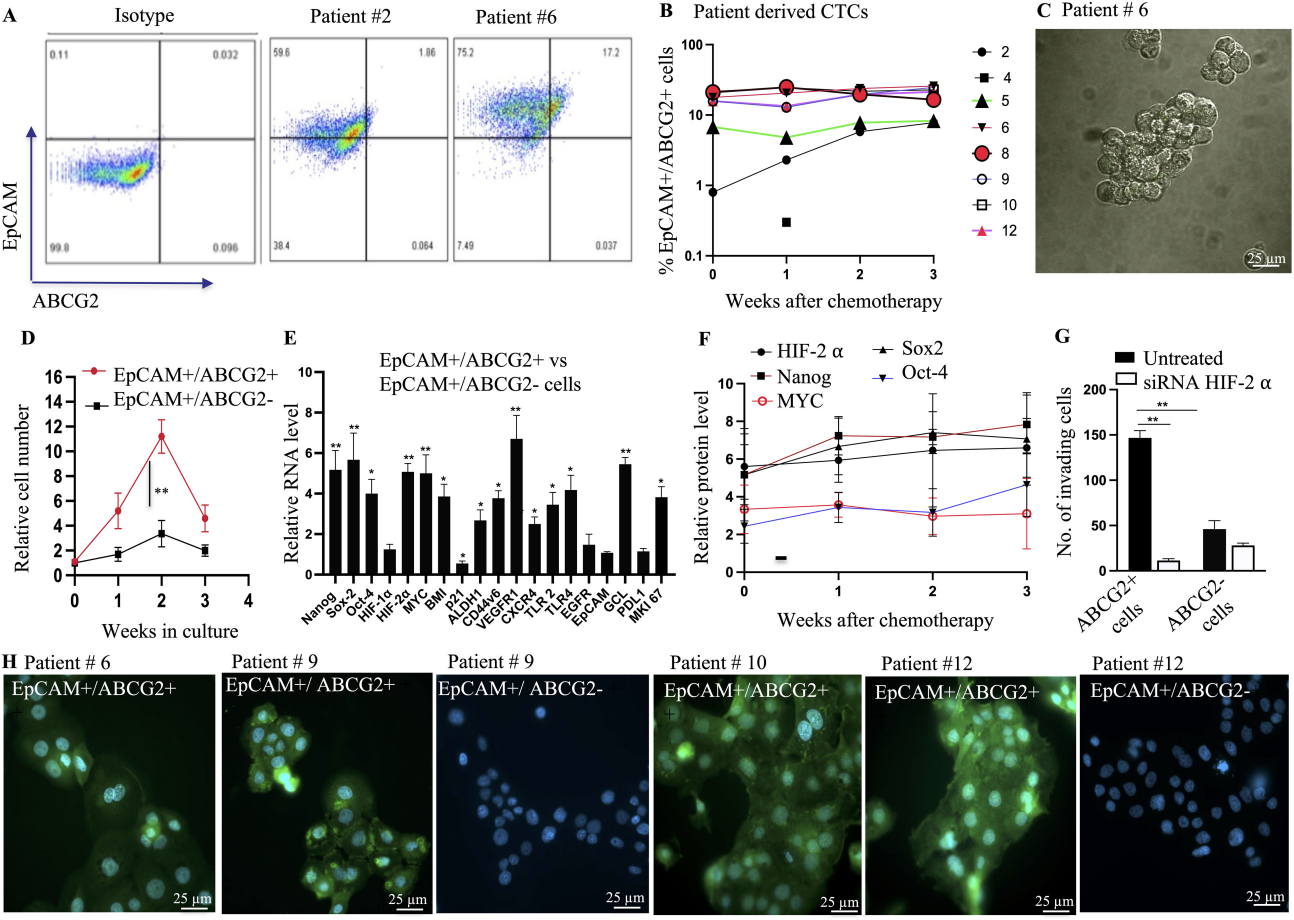
Characterization of EpCAM+/ABCG2+ CTCs with TSD phenotype. **(A)** Flow cytometry analysis reveals the presence of EpCAM+/ABCG2+ cells within multicellular spheroids derived from patient peripheral blood mononuclear cells (PBMCs) after chemotherapy. **(B)** The percentage of EpCAM+/ABCG2+ cells within these spheroids was quantified for various patients (weeks after chemotherapy). **(C)** Phase-contrast microscopy image shows isolated EpCAM+/ABCG2+ cells from a patient sample (patient #6) for further analysis. Magnification 20X **(D)** EpCAM+/ABCG2+ cells displayed increased growth compared to EpCAM+/ABCG2- cells (patients 6, 8, 9, 10, 12) when grown under low-oxygen (2% O2) conditions in serum-free media. **(E)** Real-time PCR confirmed the expression of genes associated with the TSD phenotype in sorted EpCAM+/ABCG2+ cells from multiple patients (n=5, patients 6, 8, 9, 10, 12). **(F)** ELISA measured levels of TSD-related proteins in EpCAM+/ABCG2+ cells grown in serum-free media for two weeks (pooled from patients). No significant changes in protein levels were observed across cells isolated from different time points (weeks 0-3) after chemotherapy. **(G)** Boyden chamber assay assessed the invasive potential of EpCAM+/ABCG2+ cells with and without silencing of HIF-2α (a hypoxia-inducible factor) in cells from multiple patients (n=5, patients 6, 8, 9, 10, 12). **(H)** Immunofluorescence microscopy demonstrated pimonidazole staining (a marker of hypoxia) in EpCAM+/ABCG2+ cells from various patients (patients 6, 9, 10, 12). Data are presented as mean ± SEM. Statistical significance was determined using Student’s t-test **(B**, **F**, **G)**, One way ANOVA **(D**, **E)**. (*p<0.05, **p<0.01).

Since the TSD phenotype is enriched in cells expressing ABCG2 (12), we analyzed the spheroids using flow cytometry to identify the EpCAM+/ABCG2+ population. This analysis revealed 8-18% of EpCAM+/ABCG2+ cells in the spheroids from patients #6, 8, 9, 10, and 12 (**Figures 2A, B**). To further investigate the TSD phenotype characteristics, we isolated EpCAM+/ABCG2+ cells from these patients’ spheroids with a purity of 90-92% (12). These sorted cells were then evaluated for their TSD phenotype using several assays. First, we evaluated the self-sufficiency and transient expansion by growing the cells in the serum-free media for two weeks under 2% oxygen; the sorted cells formed clusters on day 2 (**Figure 2C**), followed by rapid expansion for two weeks as measured by single cells out of the dissociated spheroids (**Figure 2D**). This expansion was then followed by a sharp decline in cell numbers by the third week, suggesting transient expansion, a key characteristic of the TSD phenotype. In contrast, EpCAM+/ABCG2- cells did not exhibit this rapid expansion when grown in the serum-free media and hypoxia (**Figure 2D**).

Second, the sorted EpCAM+/ABCG2+ cells of patients 6, 8-10, and 12 expressed genes specific to the TSD phenotype (**Figure 2E**) including the core transcription factors associated with the MYC-HIF-2alpha stemness pathway (20), the CSC markers of HNSCC including CD44v6, ALDH1 and BMI, the immunosuppressive molecules PDL1, as well as the autocrine growth factor receptors VEGFR1, CXCR4, EGFR, and TLR 2/4. The conditioned media of these cells secreted the corresponding growth factors VEGF, SDF-1alpha, EGF, and the TLR2/4 ligand HMGB1 (**Supplementary Figure S2A**) indicating their self-sufficiency potential. Third, self-sufficiency was associated with the maintenance of stemness throughout the two weeks of transient expansion (**Supplementary Figure S2B**). Notably, no significant changes were observed in protein levels of HIF-2alpha, MYC, Nanog, Sox-2, and Oct-4 in the EpCAM+/ABCG2+ cells across weeks 0-3 of chemotherapy (**Figure 2F**) indicating a continuous process of CTC mobilization in these patients. Fourth, the sorted cells exhibited marked invasion in a Boyden chamber assay, and the migration/invasion appeared dependent on HIF-2 alpha (**Figure 2G**). Finally, the conditioned media of the cells exhibited high levels of GSH, and squalene, two endogenous antioxidants, plus exhibited cytoprotection of EpCAM+/ABCG2- cells from the platinum-induced toxicity (**Supplementary Figures S2C, D**). These findings suggest that EpCAM+ ABCG2+ CTCs isolated from patients 6, 8, 9, 10, and 12 possess characteristics of the TSD phenotype including altruistic behavior. Therefore, these cells are the candidate circulating altruistic cancer stem cells (A-CSCs) with TSD phenotype.

### EpCAM+/ABCG2+ CTCs exhibit hypoxic phenotype

Considering that the reprogramming of CSC to the TSD phenotype may occur in the hypoxic niche of tumors (15), and these cells may be mobilized to the circulation, we evaluated the hypoxic phenotype of the EpCAM+/ABCG2+ CTCs. In patients 6, 9, 10, and 12, pimonidazole was administered orally (500 mg/m^2^) 48 h before blood collection in the third week of chemotherapy. Patient 8 was not included as we did not receive consent for oral pimonidazole treatment. The isolated PBMCs (collected from 10 mL of blood and grown *in vitro* for two days in the ICM) were directly subjected to immunomagnetic sorting for the EpCAM+/ABCG2+, and EpCAM+/ABCG2- cells. The sorted cells were incubated in collagen-coated chamber slides for 4-6 h to perform immunofluorescence (IF) for pimonidazole. The IF staining of the sorted cells is shown in **Figure 2H**, which indicates that only EpCAM+/ABCG2+ cells showed cytoplasm staining for pimonidazole. Noted that we could not obtain EpCAM+/ABCG2- cells from the patient # 6 and 10. Next, taking advantage of the IF images, we directly counted the EpCAM+/ABCG2+ cells per microscopic field and calculated the total number of cells per milliliter of blood as previously described (15). We found significantly higher numbers of EpCAM+/ABCG2+ cells in the circulation (434, 667, 1124, and 840 cells per milliliter of blood in patients 6, 9, 10, and 12, respectively) than EpCAM+/ABCG2- cells (0, 98, 0 and 82 cells per milliliter of blood in patients 6, 9, 10, and 12 respectively). These results suggest that EpCAM+/ABCG2+ CTCs, but not EpCAM+/ABCG2- CTCs, likely originated from hypoxic regions within the tumor before entering the peripheral blood.

### Investigating the source of CTCs exhibiting TSD phenotype in primary tumors

Building on the hypothesis that dormant EpCAM+/ABCG2+ CSCs residing in the hypoxic niche of tumors reprogram to the TSD phenotype and become CTCs, we investigated the presence of these cells in primary tumors. We characterized the primary tumor cells isolated from a subset of patients (2, 4, 6, 10, 12, and 14, **Supplementary Table S1**, **Supplementary Figure S3**), as these were the only tumor samples collected after oral pimonidazole administration (for hypoxic niche localization) and containing sufficient viable cells (~5x10^7). We categorized patients based on the presence of TSD+ CTCs: the TSD-positive CTC group (patients 6, 10, and 12) and the TSD-negative CTC group (patients 2, 4, and 14).

**Figure 3:**
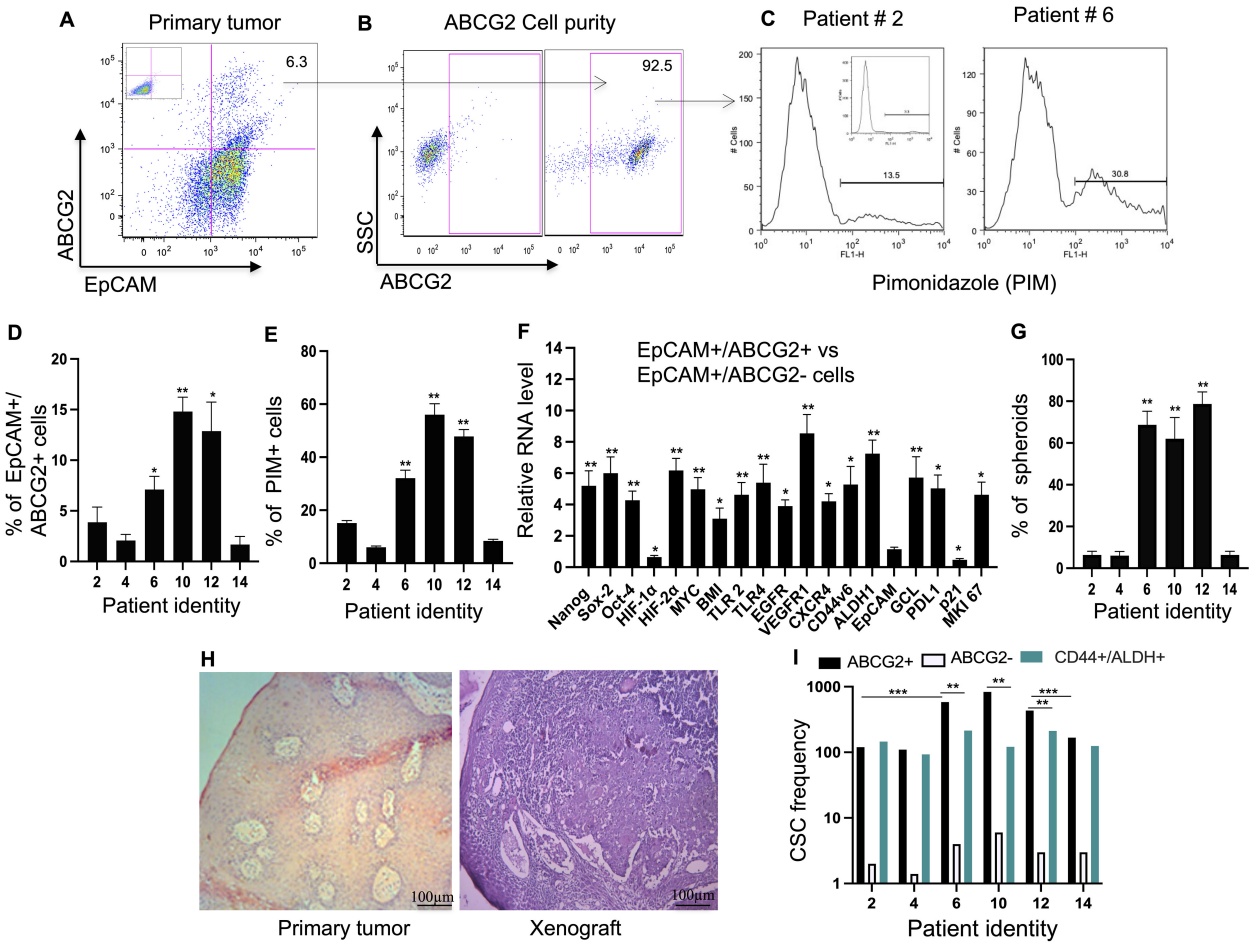
Identification of TSD+ EpCAM+/ABCG2+ cell Phenotype in Primary HNSCC. **(A)** Flow cytometry analysis demonstrates the presence of EpCAM+/ABCG2+ cells in a patient sample (patient #6, see **Supplementary Table 1**). **(B)** Purity of the immunomagnetically sorted ABCG2+ cells was assessed using flow cytometry. **(C)** Representative flow cytometry data shows pimonidazole (PIM, a hypoxia marker) staining in EpCAM+/ABCG2+ cells. **(D, E)** Quantification of data from panels **(A, C)** (percentage of EpCAM+/ABCG2+ cells and their PIM staining). The mean value of patient 2, 4 and 14 was used to compare the data. **(F)** Real-time PCR analysis revealed differential gene expression patterns associated with the TSD+ phenotype in sorted EpCAM+/ABCG2+ cells compared to EpCAM+/ABCG2- cells from patients 6, 10, and 12. **(G)** EpCAM+/ABCG2+ cells from specific patients (patients 6, 10, and 12) formed significantly more spheroids compared to cells from other patients (patients 2, 4, and 14).  (Student's t-test; **p < 0.01). **(H)** Hematoxylin and eosin (H&E) stained sections show a primary tumor (patient #10) and the corresponding xenograft derived from transplanted EpCAM+/ABCG2+ cells in NOD/SCID mice. **(I)** CSC frequency per 10^5^ tumor cells as performed by in vivo limiting dilution assay. Data in panels **(F–G)** are presented as mean ± SEM. One Way Anova was used, with *p<0.05, **p<0.01, ***p<0.001 indicating statistical significance.

The dissociated primary tumor cells contained 40-60% EpCAM+ cells (**Figure 3A**; **Supplementary Figures S3A, B**) consistent with previous reports (12, 26, 27). Following immunomagnetic sorting, a highly enriched population (~92% purity) of EpCAM+/ABCG2+ CSCs was obtained (**Figure 3B**). These isolated cells then underwent further analysis: flow cytometry with pimonidazole staining to assess hypoxia (**Figure 3C**), characterization for dormancy, and the TSD phenotype.

Interestingly, the EpCAM+/ABCG2+ CSC population exhibited variations depending on the patient. The TSD positive CTC group harbored a cumulative 6.7-fold higher percentage of these EpCAM+/ABCG2+ CSCs compared to the TSD negative CTC group (p<0.05; **Figure 3D**). This suggests a potential link between the abundance of EpCAM+/ABCG2+ CSCs and the presence of TSD+ CTCs.

Further analysis using pimonidazole revealed a potential association between hypoxia and the characteristics of EpCAM+/ABCG2+ CSCs. A significantly higher proportion (30-60%) of these cells were hypoxic in the TSD-positive CTC group compared to less than 10% in the TSD-negative CTC group (**Figure 3E**). Notably, the bulk tumor cells obtained from the TSD negative CTC group displayed a 6-7-fold lower proportion of pimonidazole-positive cells compared to the TSD positive CTC group (p<0.02; **Supplementary Figure S4A**). Furthermore, association study suggests a potential link between the hypoxic TME and the enrichment of TSD+ CSCs in the TSD-positive CTC group (r2 = 0.86; p<0.03).

To explore this link further, we performed gene expression analysis. EpCAM+/ABCG2+ CSCs from the TSD-positive CTC group exhibited high expression of genes associated with the TSD phenotype (**Figure 3F**), whereas those from the TSD-negative CTC group displayed lower expression of TSD phenotype genes (**Supplementary Figure S4B**). However, the latter group showed a 3-fold higher expression of p21, a marker associated with dormancy, and a 6-fold lower expression of the proliferation marker Ki-67 (**Supplementary Figure S4B**). These findings align with the results from Ki-67 staining and spheroid formation assays. Patients 6, 10, and 12 (TSD-positive CTCs) displayed a more aggressive phenotype with higher Ki-67 positivity (**Supplementary Figure S4C**) and robust spheroid formation in culture (**Figure 3G**). Conversely, patients 2, 4, and 14 (TSD-negative CTCs) exhibited a dormant CSC population with minimal Ki-67 positivity and fewer spheroids (**Figure 3G**; **Supplementary Figure S4C**). The gene expression study also revealed an interesting difference in the MYC-HIF-2alpha stemness pathway, which is potentially linked to the TSD phenotype. EpCAM+/ABCG2+ cells of the TSD-negative CTC group downregulated the genes involved in the MYC-HIF-2alpha stemness pathway but upregulated HIF-1alpha (**Supplementary Figure S4B**). This suggests a distinct activation pattern within the HIF pathway between the two groups. Moreover, the expression of CXCR4, a chemokine receptor, was fourfold higher in the CTC-negative *versus* positive cells (**Supplementary Figure S4B**). These data, along with the gene expression data of p21 and Ki-67, may suggest a potential gene signature of the putative dormant CSC phenotype, as we explore later in the paper.

*In vivo* limiting dilution assays confirmed the self-renewal capacity of the isolated EpCAM+/ABCG2+ cells (**Figures 3H, I**; **Supplementary Table 2**), suggesting their CSC phenotype. Notably, patients 6, 10, and 12, who displayed TSD+ CTCs and a higher abundance of EpCAM+/ABCG2+ CSCs, exhibited a two- to sixfold higher CSC frequency compared to patients 2, 4, and 14. Furthermore, EpCAM+/ABCG2+ CSCs from patients 6, 10, and 12 displayed a twofold higher tumorigenicity compared to CD44+/ALDH+ cells (**Figure 3I**; **Supplementary Table S2**), another established CSC population isolated from the same tumors (28). This finding highlights that EpCAM+/ABCG2+ cells are enriched in the CSCs and that the TSD-positive CTC group displayed a more aggressive phenotype with a higher enrichment of CSCs. In contrast, the EpCAM+/ABCG2+ CSCs of the TSD-negative CTC group displayed a dormant phenotype with a distinct gene expression of high p21 and low Ki-67 as well as a higher level of CXCR4.

### EpCAM+/ABCG2+ CSCs exhibit altruistic behavior

To further decipher the TSD+ phenotype of EpCAM+/ABCG2+ CSCs, we investigated their behavior in serum-free media and hypoxia (2% oxygen), mimicking the stressful TME. We have characterized this *in vitro* assay to test the self-sufficiency (ability to grow and maintain stemness without nutrition and growth factor supplements as measured by sphere formation and stemness marker expression) and altruism (supporting neighboring cells by sacrificing self-fitness as measured by the cytoprotective capacity of the conditioned media) behavior of mesenchymal altruistic stem cells (M-ASCs) (23) as well as the hypoxia-induced TSD phenotype of SCC-25 cells (12).

**Figure 4:**
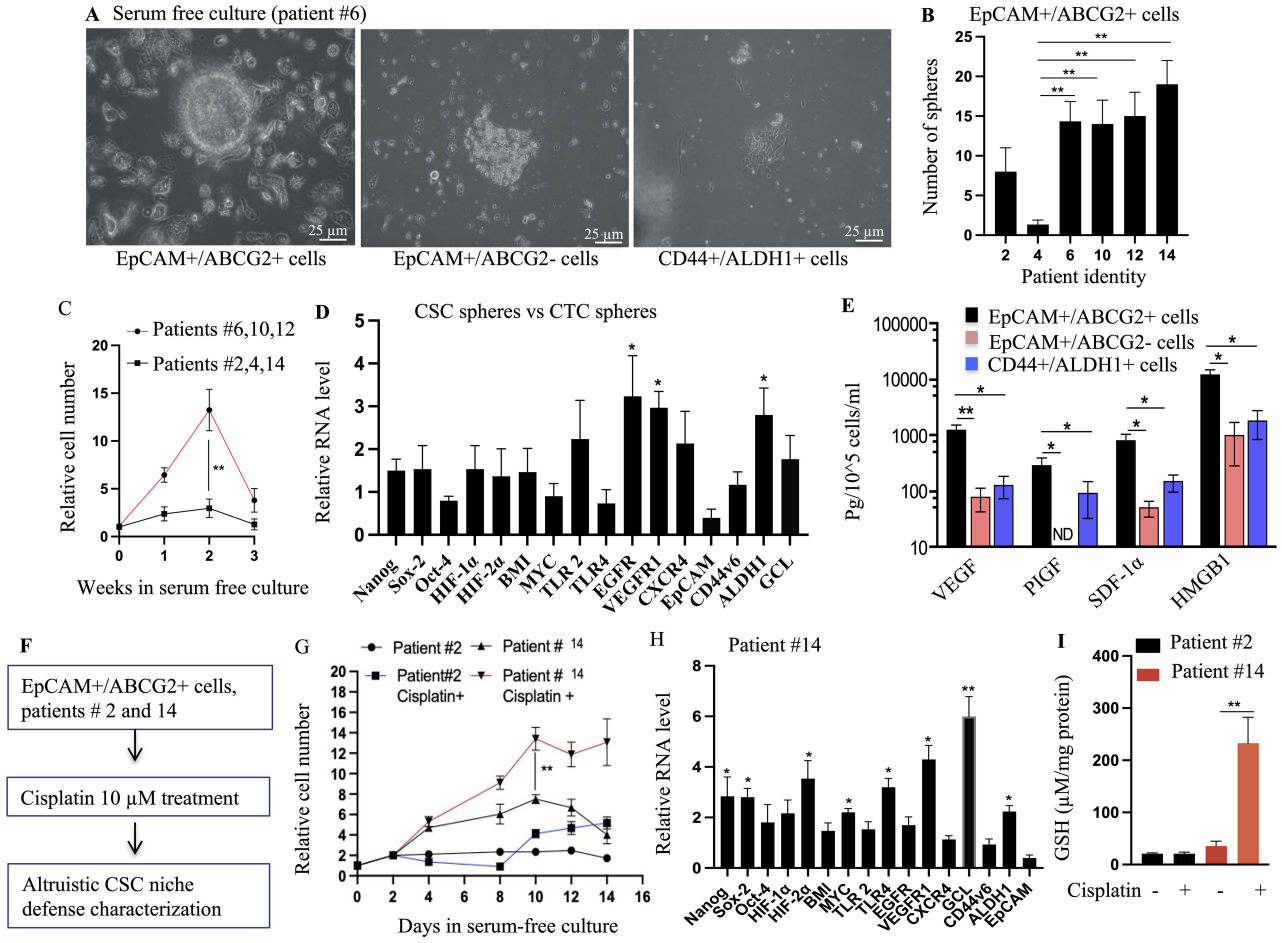
Identification of TSD phenotype in primary HNSCC: self-sufficiency and niche defense. **(A)** EpCAM+/ABCG2+ cells from patient #6 formed tumorospheres when grown under 2% oxygen in serum-free media for 8 days, while EpCAM+/ABCG2- and CD44+/ALDH+ cells from the same tumor did not. **(B)** Quantification of the number of tumorospheres formed by EpCAM+/ABCG2+ cells from different patient tumors. **(C)** The expansion of EpCAM+/ABCG2+ cells in the tumorospheres. The tumorospheres were dissociated and subjected to flow cytometry analysis to obtain the total number of EpCAM+/ABCG2+ cells. **(D)** EpCAM+/ABCG2+ cells isolated from the tumorospheres exhibit TSD+ gene expression pattern, similar to EpCAM+/ABCG2+ cells of CTC spheroids. **(E)**  EpCAM+/ABCG2+ cells obtained from tumorospheres were cultured in 2% oxygen+serum-free media (day 10) to measure the secretion of growth factors involved in self sufficiency of TSD+ phenotype. Conditioned media (CM) was collected from these cells to test altruistic niche defense behavior. **(F)** Schematic overview of the CSC niche defense experiment design. **(G)** Patient-derived EpCAM+/ABCG2+ cells were grown in injured conditioned media and then treated with cisplatin (10 uM for 48 hours). Cell survival was measured using trypan blue exclusion. **(H)** Real-time PCR analysis compared gene expression profiles of EpCAM+/ABCG2+ cells from patient #14 treated with cisplatin versus untreated cells (day 10). **(I)**  Quantification of Glutathione (GSH) in the EpCAM+/ABCG2+ cells obtained from day 10 culture shown in **Figure 4G**. The cells were grown in serum free media for 48 hours to measure GSH. Data in panels **(B–E)**, and **(G–I)** are presented as mean ± SEM. Student's t-test was used in **(C**, **G**, **I)**. One way ANOVA was used in **(B**, **D**, **E**, **H)**,  with *p<0.05, **p<0.01, ***p<0.001 indicating statistical significance.

Importantly, to ensure that the observed effects were specific to EpCAM+/ABCG2+ CSCs, we included control groups: CD44+/ALDH+ CSCs and EpCAM+/ABCG2- cells. Interestingly, only EpCAM+/ABCG2+ CSCs isolated from the TSD-positive CTC group (patients 6, 10, and 12) formed 3D spheroids under these stressful conditions, with a six- to eightfold proliferation between the first and second week of culture (**Figures 4A–C**).

Furthermore, conditioned media obtained from these spheroids (second week) protected the neighboring EpCAM+/ABCG2- cells grown under starvation (**Supplementary Figure S5A**). This intriguing observation suggests the secretion of protective factors by these cells while maintaining stemness. Supporting this notion, the cells obtained from the spheroids expressed genes and secreted factors associated with the TSD phenotype (**Figures 4D, E**). In contrast, the control groups, EpCAM+/ABCG2- and CD44+/ALDH+ CSCs, neither formed spheroids (**Figure 4A**) nor exhibited the TSD phenotype even in nutrient-rich conditions (**Supplementary Figure 5B**).

While these CSCs exhibit altruistic behavior by secreting protective factors, a key question remains—is there a self-sacrificing element to this altruism? To address this, we focused on the fate of the spheroids during the period when their proliferation decreased (between weeks 2 and 3). We drew parallels to established models of altruistic behavior in mesenchymal stem cells and embryonic stem cells (13, 23), where these cells undergo apoptosis (programmed cell death) while simultaneously protecting neighboring cells through secreted factors in the conditioned media. Intriguingly, our findings mirrored these observations. The EpCAM+/ABCG2+ CSCs within the spheroids displayed marked activities of apoptosis, which can be inhibited by treating the cells with a p53 inhibitor pifithrin alpha (**Supplementary Figure S5C**). This suggests that these cells might be undergoing self-sacrifice in a p53-dependent manner. Furthermore, the conditioned media from these potentially self-sacrificing spheroids allowed the neighboring EpCAM+/ABCG2- cells to proliferate even during serum starvation (**Supplementary Figure S5D**). Thus, the EpCAM+/ABCG2+ CSCs, when grown in serum-free media plus hypoxia, not only exhibit an altruistic ability to protect neighboring cells but might also do so through a self-sacrificing mechanism, further aligning their behavior with the characteristics of TSD phenotype. Therefore, to acknowledge these functional similarities, we propose the term altruistic cancer stem cells (A-CSCs) for CSCs displaying the TSD phenotype.

### The idea and evidence of reawakening CSCs with stress memory

As per our working hypothesis, we expect all EpCAM+/ABCG2+ CSCs to exhibit altruistic behavior when exposed to a serum-free and hypoxic microenvironment that mimics the stressful microenvironment of intermittent hypoxia and oxidative stress prevalent in the tumor. However, the CSCs of the CTC-negative group (patients 2, 4, and 14) remained dormant under these conditions (**Supplementary Figure S5E**). This observation contrasted with the activation observed in the TSD+ CTC group and challenged our initial hypothesis of a uniform altruistic response from all EpCAM+/ABCG2+ CSCs.

To reconcile this surprising discrepancy, we employed Satavata Tarka, a unique suppositional reasoning practiced in our community-based participatory action research (CBPAR) framework (18, 19). This Tarka encourages the emergence of novel “dharana” (hypotheses) through logical reasoning and collaborative discussion. This approach led us to hypothesize the existence of R-CSCs with a potential “stress memory” that mediate tumor self-defense. The gene expression analysis of the dormant EpCAM+/ABCG2+ CSCs from patients 2, 4, and 14 revealed high levels of CXCR4 and ALDH1 but a low expression of genes associated with the MYC-HIF-2α stemness pathway. We propose that this profile characterizes R-CSCs that “remember” past cellular stress. This concept suggests that R-CSCs might be a subpopulation of disseminated cancer cells (DCCs). They possess stem cell-like properties and the potential to remain dormant for extended periods. However, unlike typical DCCs that might require specific signals for reactivation, R-CSCs may have a “stress memory.” Due to past stress exposure (like chemotherapy exposure), R-CSCs might require an additional trigger besides the usual cues for DCC reactivation (favorable niche and reawakening dormancy signals).

Supporting this hypothesis is the distinct behavior of CSCs isolated from patient 14, who had received prior cisplatin treatment (**Figures 4F–I**). These cells exhibited the TSD phenotype only when additionally exposed to cisplatin *in vitro*, suggesting a reactivation by the chemotherapy drug (**Figure 4H**). Conversely, CSCs from patient 2, who lacked prior cisplatin treatment, remained unresponsive to cisplatin treatment (**Figure 4G**). These findings suggest the potential importance of R-CSCs in TSD+ CTC generation and treatment resistance, warranting further investigation. To further explore these concepts, we formulated key questions using another Satavata Tarka session: Are TSD+ CSCs truly altruistic or can they undergo selection for survival and acquire stable phenotypes such as R-CSCs? What is the precise link between CSC niche defense, tumor hypoxia, TSD+ CTCs, and the altruistic behavior of TSD+ CSCs?

We adapted an existing pre-clinical model (details provided below) to translate our clinical findings into a platform suitable for *in vivo* investigation.

### Pre-clinical modeling of TSD phenotype and CTC emergence in cisplatin-treated HNSCC

**Figure 5:**
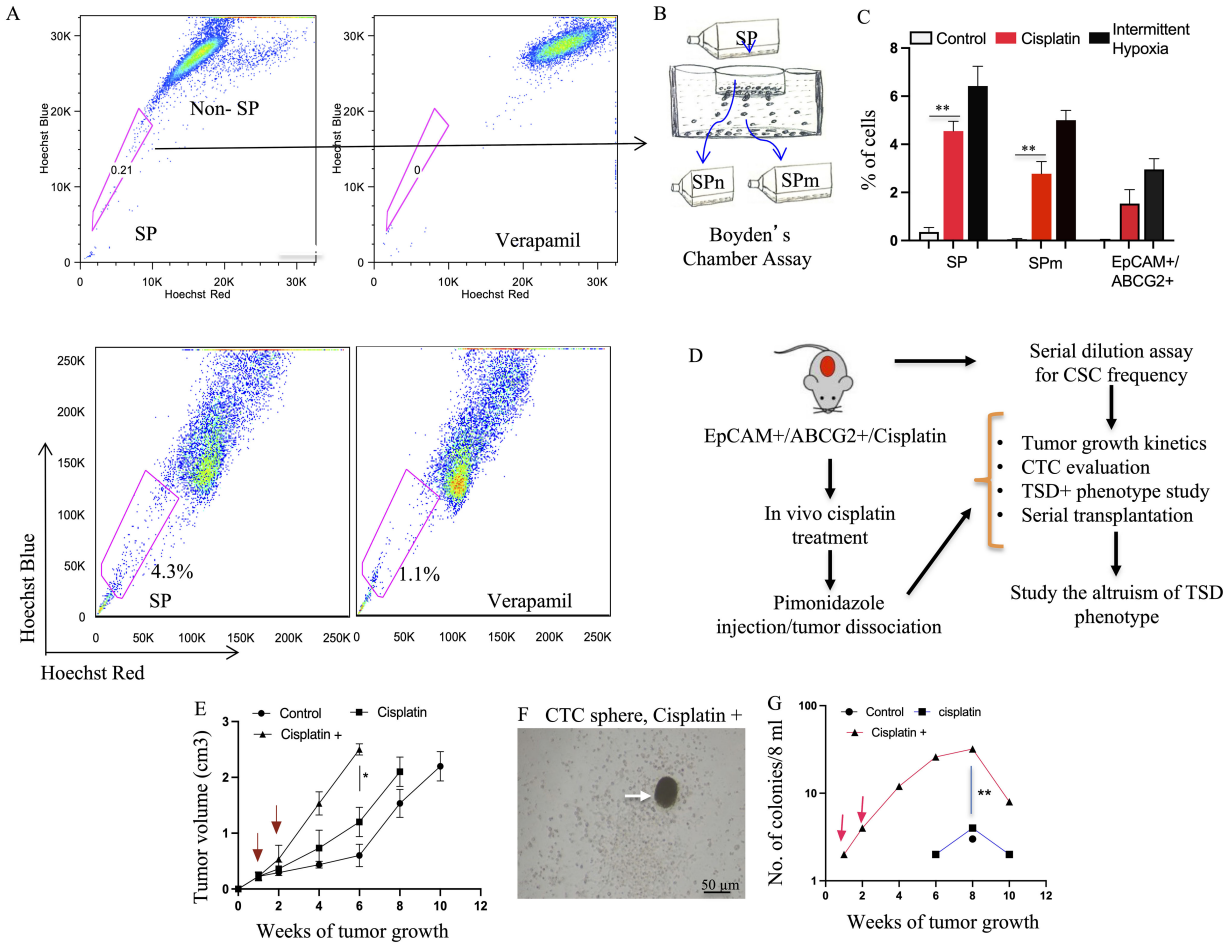
Preclinical model of cisplatin-Induced enrichment of TSD+ CSC and R-CSC. **(A)** Flow cytometry analysis reveals expansion of the side population (SP) fraction in SCC-25 cells following cisplatin treatment (10 μM, 4 days) under 2% oxygen in serum-free media, potentially enriching for R- CSCs. **(B, C)** Boyden chamber assay isolates migratory SP (SPm) cells. Cisplatin treatment increases SP, SPm, and EpCAM+/ABCG2+ cell populations compared to untreated controls. Intermittent hypoxia (hypoxia/re-oxygenation) serves as a positive control. **(D)** Schematic depicts the *in vivo* experiment design (based on Satavata Tarka) to generate R-CSCs and the TSD phenotype within EpCAM+/ABCG2+ cells. **(E)** Post-cisplatin EpCAM+/ABCG2+ cells form tumors with faster growth in NOD/SCID mice after additional cisplatin treatment (10 mg/kg, red arrow). 10,000 post-cisplatin EpCAM+/ABCG2+ cells were injected subcutaneously in mice. After one week, these mice received additional cisplatin treatment for two weeks. **(F)** Arrow shows tumorosphere obtained by culturing CD45- PBMCs of xenograft bearing mice. **(G)** The number of CTC spheroid increases in the cisplatin+ group as the tumor grow. For **(C, E, G)**, data represent +/- SEM. One way ANOVA was used in **(C)**. Student’s t-test was used in **(E, G**), *p<0.05, **p<0.01.

To address the knowledge gaps identified through Satavata Tarka (as discussed above), we adapted a previously established pre-clinical model that utilizes chemotherapy to induce a state of tumor stemness. In this model, brief cisplatin treatment under serum starvation expands a dormant side-population (SP) cell population, giving rise to migratory SP (SPm) cells exhibiting a tumor stemness phenotype (9). Here we adapt this model using the human HNSCC cell line SCC-25, known to exhibit the TSD phenotype under hypoxia and oxidative stress (12). Thus, brief cisplatin treatment under low-nutrient conditions and hypoxia led to the threefold expansion of SPm cells enriched in EpCAM+/ABCG2+ phenotype (**Figures 5A–C**; **Supplementary Figures S6A, B**). Interestingly, these post-cisplatin EpCAM+/ABCG2+ cells remained quiescent in serum-free media with 2% oxygen (**Supplementary Figure S6C**), suggesting that these cells fail to reprogram to the TSD phenotype. However, these cells displayed a gene signature (**Supplementary Figure S6D**) similar to the dormant EpCAM+/ABCG2+ CSCs observed in patient 14 (as previously described in **Supplementary Figure S4C**), further confirming that these cells failed to reprogram to the TSD phenotype. We hypothesize these post-cisplatin EpCAM+/ABCG2+ cells as the candidate dormant or R-CSCs.

**Figure 6:**
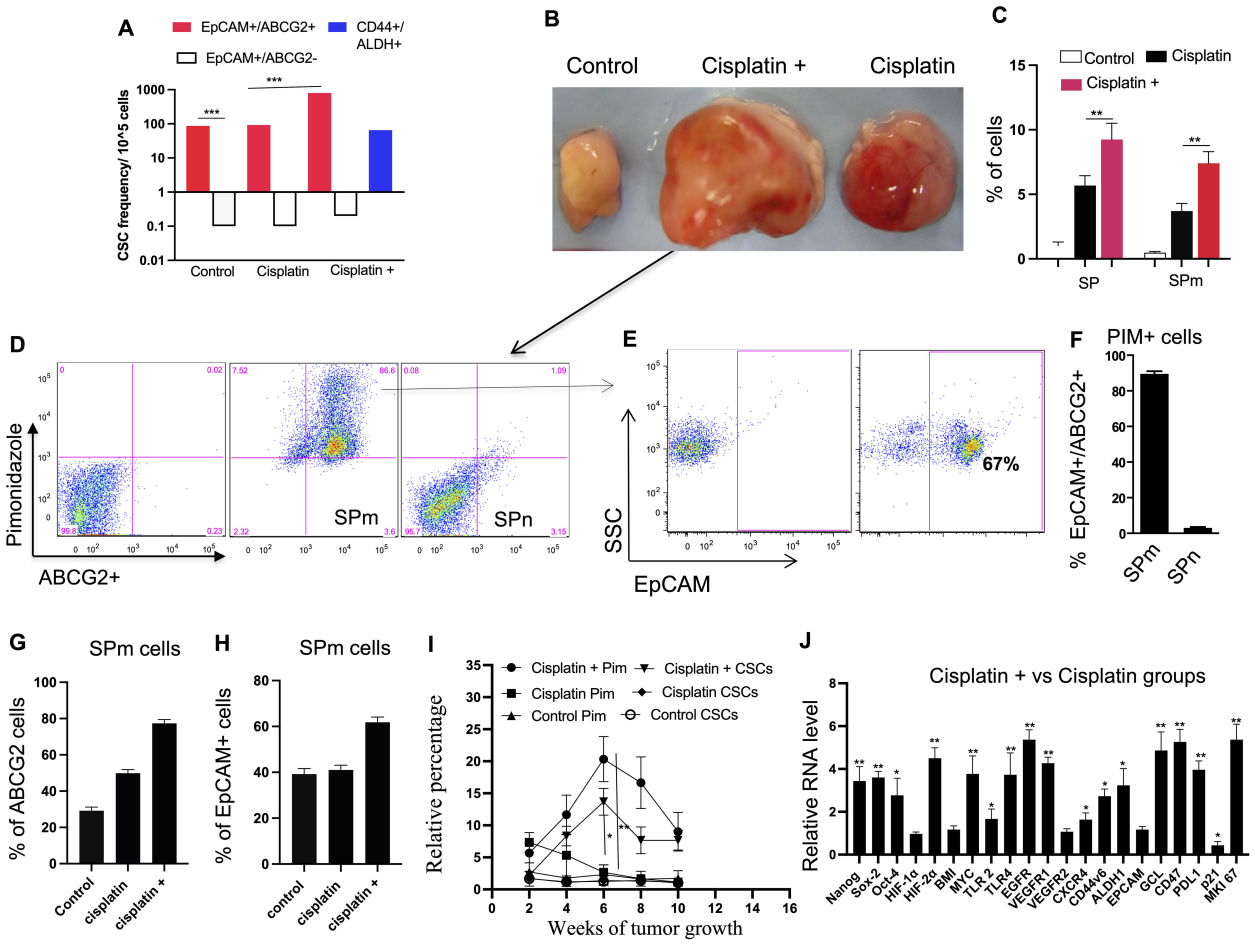
Cisplatin-induced CSC niche defense. **(A)** Limiting dilution assay (details in **Supplementary Table 3**) compares the self-renewal capacity of different cell types treated with cisplatin *in vitro* (cisplatin group), and *in vivo* (cisplatin +). **(B)** Images show tumors derived from EpCAM+/ABCG2+ cells at week 6. **(C)** Histograms show the corresponding SP and SPm fraction in the respective tumors shown in **(B)**. **(D–F)** Analysis of SP and SPm fractions from tumors identifies a population with high ABCG2 and pimonidazole (hypoxia) markers, mostly EpCAM+/ABCG2+. SPm fraction has a significantly higher percentage of ABCG2+/Pimonidazole+ cells compared to SPn. **(G, H)** The "cisplatin +" group shows increased ABCG2+ and EpCAM+ cells within the SPm fraction. **(I)** Flow cytometry analysis revealed a correlation between tumor hypoxia (pimonidazole-positive cells) and the expansion of EpCAM+ABCG2+ cells. Black denotes pimonidazole and Red denotes EpCAM+/ABCG2+ cells. **(J)** Real-time PCR reveals upregulation of TSD genes in EpCAM+/ABCG2+ cells from "cisplatin +" tumors. Data presented as mean ± SEM. Student’s t-test was used in **(C**, **F–I)**. One way ANOVA was used in **(A, J)**. *p < 0.05, **p < 0.01, ***p < 0.001.

**Figure 7:**
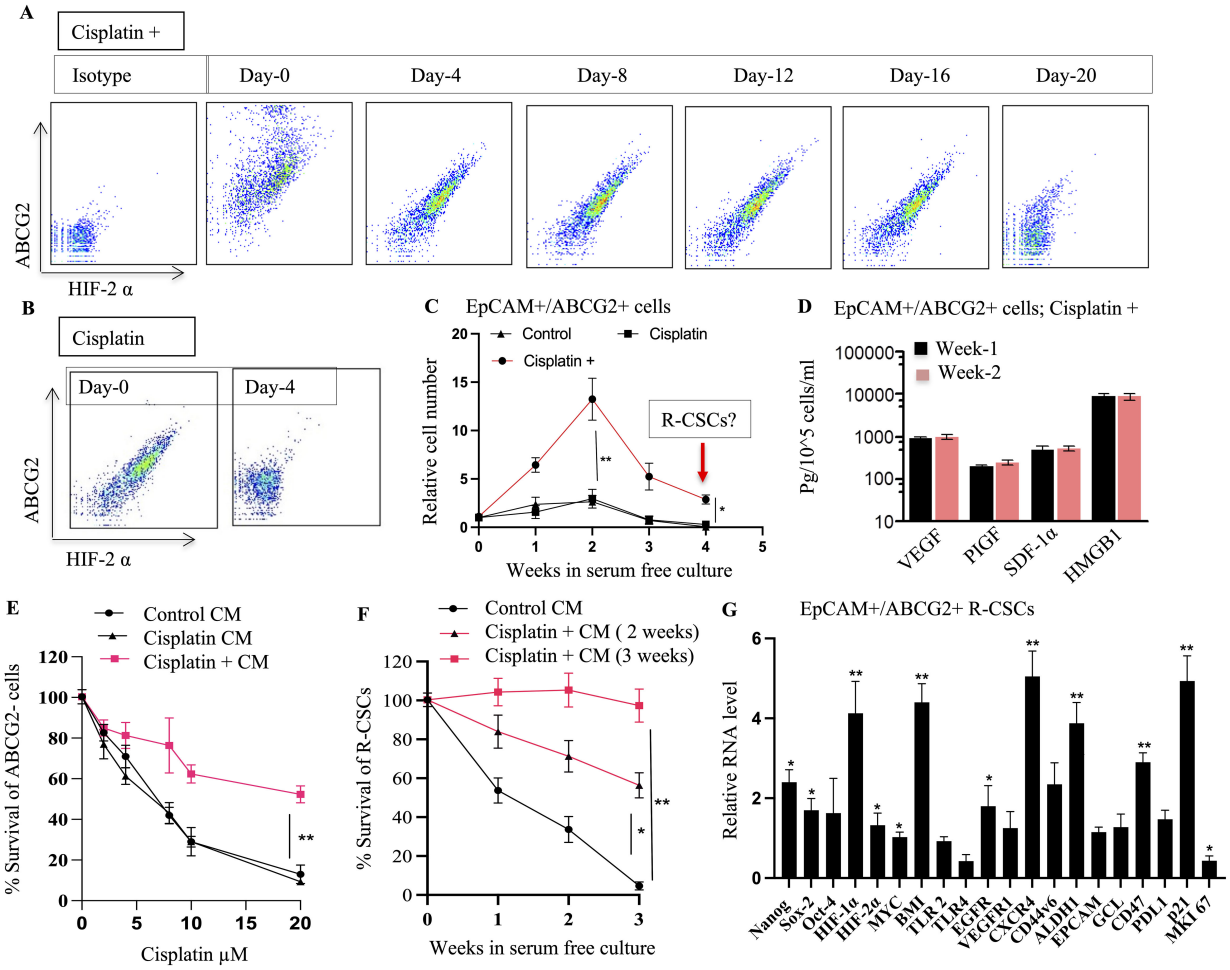
TSD Phenotype and Altruistic Behavior of Cisplatin-Treated CSCs. **(A, B)** EpCAM+/ABCG2+ cells from cisplatin+ tumors (**Figure 5**) were cultured under 2% O2 in serum-free media. Flow cytometry monitored HIF-2α and ABCG2 expression over time. Cells from the "cisplatin +" group maintained these markers, suggesting self-sufficiency and potentially an undifferentiated state. Whereas cells from the cisplatin group differentiated on day 4. **(C)** EpCAM+/ABCG2+ cells from the "cisplatin +" group displayed rapid growth even in deprived conditions (serum-free media, 2% O2) suggesting self-sufficiency. **(D)** These proliferating EpCAM+/ABCG2+ cells (week 1 and week 2) maintained a secretory phenotype, suggesting they exhibit autocrine and paracrine functions. **(E)** Conditioned media obtained from EpCAM+/ABCG2+ cells of cisplatin+ group protected the EpCAM+/ABCG2- cells treated with cisplatin. **(F)** Conditioned media obtained from EpCAM+/ABCG2+ cells (2nd and third week) protected R-CSCs (EpCAM+ABCG2+ cells collected on 4th week as shown in **(C)** grown in 2% O2+serum free media. **(G)** Gene expression analysis of R-CSCs (EpCAM+ABCG2+ cells collected on 4th week as shown in **(C)** is showing high expression of dormancy genes; p21, BMI, CD47. Data presented as mean ± SEM. One way ANOVA was used in **(C**, **D**, **G)**; Linear regression analysis for panels **(E, F)**. *p < 0.05, **p < 0.01.

We then used these post-cisplatin-treated EpCAM+/ABCG2+ cells to generate xenografts in NOD/SCID mice. These tumor-bearing animals were subjected to CTC analysis. We compared three xenograft groups (**Figure 5D**): (1)—control: evaluated if inherent CSC properties of dormant EpCAM+/ABCG2+ cells are sufficient for CTC mobilization, (2) cisplatin: evaluated if post-cisplatin EpCAM+/ABCG2+ cells (R-CSCs)-derived tumors can mobilize CTCs, and (3) cisplatin+: examined the effect of additional cisplatin treatment on CTC mobilization in mice bearing R-CSC-derived xenografts. We found that only the cisplatin+ group exhibited a twofold faster-growing tumor (reduced latency; **Figure 5E**), and CTCs were mobilized. The PBMC from the cisplatin+ group of mice displayed tumor spheroid formation in culture, peaking at 8 weeks after xenograft formation (**Figures 5F, G**). These CTCs from the 8th week xenograft contained EpCAM+/ABCG2+ cells exhibiting transient expansion and cytoprotection, suggesting a TSD phenotype (**Supplementary Figure S7**). On the other hand, the control and cisplatin group did not exhibit accelerated tumor growth (**Figure 5E**) or significant mobilization of CTCs (**Figure 5G**), suggesting a link between tumor progression and CTC mobilization. Indeed the cisplatin+ group exhibited 8.5-fold higher *in vivo* self-renewal capacity compared to the control group (**Figure 6A**). This is associated with 1) a marked increase in tumor size and SPm cell expansion (**Figures 6B, C**). 2) Enrichment of EpCAM+/ABCG2+ cells in the pimonidazole-positive SPm cells (**Figures 6D–H**). 3) Linear increase of these EpCAM+/ABCG2+ cells and tumor hypoxia between the 2-8th weeks of tumor growth (**Figure 6I**). 4) Expression of TSD phenotype-associated genes by these cells (**Figure 6J**). 5) Activation of the MYC-HIF-2alpha stemness pathway in these cells (**Supplementary Figures S8A–C**). 6) Subpopulation of EpCAM-/ABCG2+ cells expressing EMT markers, indicating potential plasticity (**Supplementary Figure S8D**) 7) These EpCAM+/ABCG2+ cells derived xenograft growth can be inhibited by treating mice with Axitinib and Erlotinib (**Supplementary Figure S8E**) indicating that VEGFR1 and EGFR may be involved with the cisplatin-induced tumor stemness switch, consistent with our previous findings (9). 8) EpCAM+/ABCG2+ cells isolated from the cisplatin+ group tumors exhibited self-sufficiency, transient expansion, and secretion of autocrine growth factors with cytoprotective effects (**Figures 7A–E**).

Furthermore, our investigation revealed a distinct R-CSC population within the TSD+ EpCAM+/ABCG2+ CSC population. Notably, this R-CSC population demonstrated a remarkable growth even during the 3rd to 6th week of *in vitro* culture in serum-free media. This extended growth was observed when the R-CSC population was maintained in conditioned media from the dying TSD+ EpCAM+/ABCG2+ cells (**Figures 7C, F**). Remarkably, the R-CSCs exhibited a gene expression profile (**Figure 7G**), which is similar to the *in vitro* cisplatin-treated EpCAM+/ABCG2+ cells (**Supplementary Figure S6D**).

This adapted pre-clinical model indicates that cisplatin treatment can reawaken a dormant population of EpCAM+/ABCG2+ CSCs in SCC-25 cells *in vitro*. When these R-CSCs were engrafted in mice and treated with cisplatin *in vivo*, the CSC niche defense mechanism was activated. Consequently, a subpopulation of the EpCAM+/ABCG2+ CSCs within the xenograft reprogrammed to the TSD phenotype rapidly expanded and mobilized into the circulation as CTCs. Both the CSCs within the tumor and the mobilized CTCs exhibited a hypoxic signature.

These findings align with our clinical observations of pimonidazole-positive TSD phenotype cells in primary tumors and circulating EpCAM+/ABCG2+ cells, suggesting the potential relevance of the pre-clinical model.

### TSD+ CSCs: altruism or selfishness?

**Figure 8:**
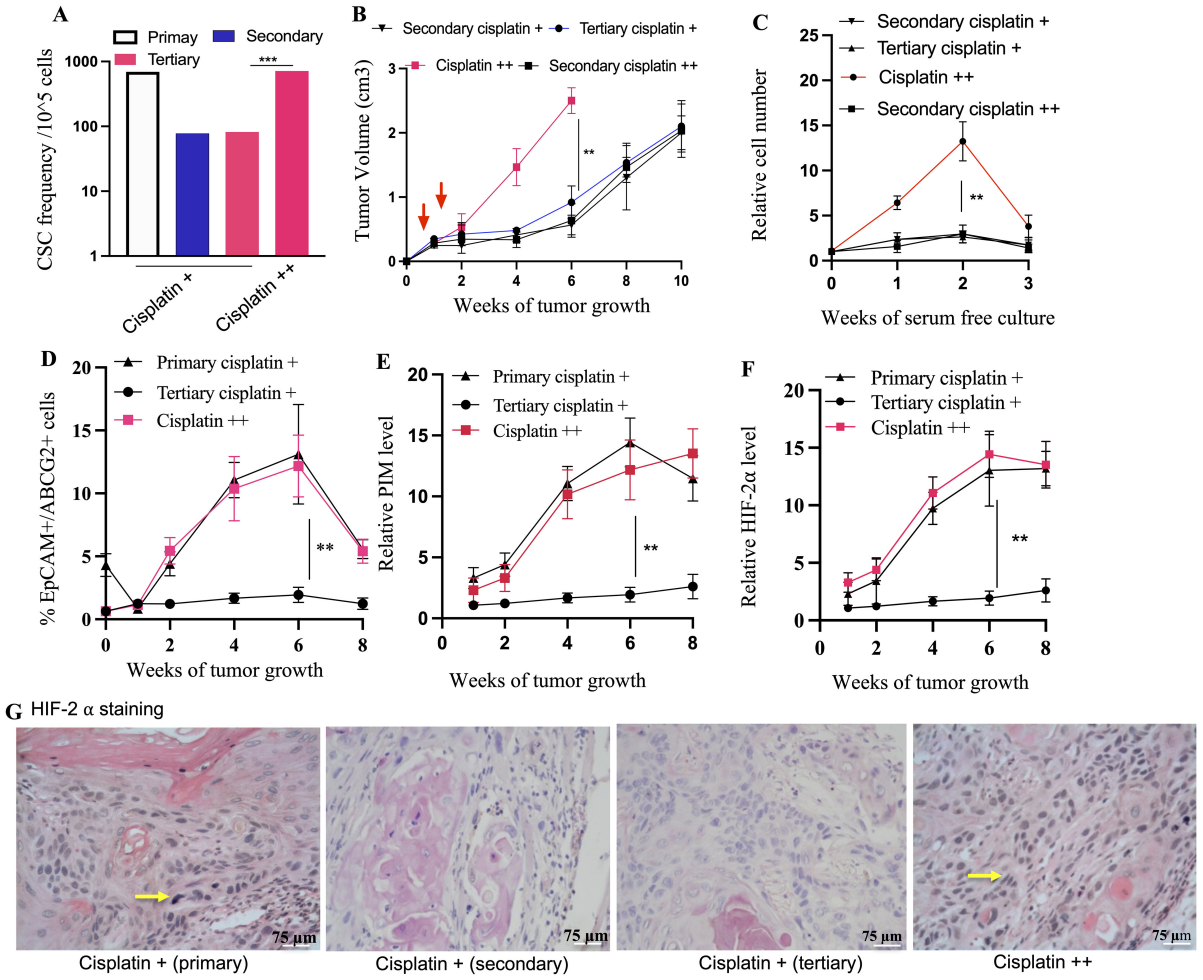
Transient TSD+ phenotype and the reawakening of dormant R-CSCs. Serial transplantation reveals decreased CSC frequency **(A)** in secondary/tertiary tumors compared to primary, suggesting the transient state of the TSD+ EpCAM+/ABCG2+ CSCs. Cisplatin treatment of tertiary tumors (cisplatin++) triggers a rebound in CSC frequency **(B)** Faster tumor growth in cisplatin++ group. Tumor cells from these group injected to mice to develop secondary cisplatin++ tumor. **(C)** rapid increase in the percentage of the EpCAM+/ABCG2+ cells obtained from tumor xenograft and grown in 2% O2+serum free media. **(D–F)** Xenograft exhibit increased number of TSD+CSCs (EpCAM+/ABCG2+ cells) exhibiting PIM and HIF2alpha during the tumor growth. It shows a decrease of pimonidazole binding and HIF-2α protein levels in cisplatin + tertiary tumors compared to the primary, and reawakened (cisplatin ++) tumors. **(G)** Immunohistochemistry is showing Hif2alpha expression in EpCAM+/ABCG2+ cells derived xenografts. Yellow arrow indicates the Hif2alpha positive cells: Data represent ± SEM, three independent experiments, Student’s t test. *p<0.05, **p<0.01.

To address whether TSD+ CSCs of the pre-clinical model are altruistic or selfish, serial transplantation experiments were conducted to track the fate of TSD+ EpCAM+/ABCG2+ CSCs across multiple generations. While CSCs can maintain a stable frequency across generations (20), mutated CSCs will alter the frequency and, therefore, self-identity of a given tumor (14). Simultaneously, the mobilization of potential TSD+ CTCs to the metastatic niche site such as bone marrow was studied to find out whether the TSD+ CSCs may act selfishly by developing rapid metastatic tumor growth. We found that TSD+ CSCs were reversible in secondary and tertiary tumors (**Figure 8A**). The CSC frequency in TSD+ EpCAM+/ABCG2+ generated xenografts which returned to baseline levels in the subsequent third generation (**Figure 8A**), similar to non-cisplatin-exposed parental SCC-25 cells (**Supplementary Table S3**). This data confirms that the cisplatin treatment-induced TSD+ phenotype was a reversible, non-genetic phenomenon and therefore, not selfish.

Intriguingly, a subpopulation of EpCAM+/ABCG2+ CSCs appeared to transition to a dormant state: the R-CSCs. These R-CSCs seem to retain a “memory” of the TSD phenotype’s stress response to cisplatin. This is evidenced by the rapid expansion of TSD+ CSCs, their self-sufficiency, and high HIF-2α expression observed in tumors derived from these R-CSCs upon re-exposure to cisplatin (the cisplatin++ group, **Figures 8A–G**). Moreover, the cisplatin++ group (cisplatin treatment of tertiary tumors) exhibited a growth pattern similar to the primary cisplatin+ group. Both tumor hypoxia and the number of TSD+ EpCAM+/ABCG2+ CSCs were increased significantly in the cisplatin++ group between the 2nd and 8th weeks of tumor growth (**Figures 8F–G**). This observation is supported by a strong correlation coefficient of 0.89 (*p* < 0.001), suggesting a robust association between hypoxia and the expansion of TSD+ EpCAM+/ABCG2+ CSCs. Thus, the tertiary transplant tumors retained a cisplatin-induced stress memory in a non-genetic manner, without undergoing a selection process. It was noted that EpCAM+/ABCG2- cells as well as CD44+/ALDH1+ CSCs obtained from the tertiary transplant cisplatin+ group did not exhibit rapid growth upon cisplatin treatment (**Supplementary Table S3**), suggesting that these cells were not enriched in R-CSCs.

**Figure 9:**
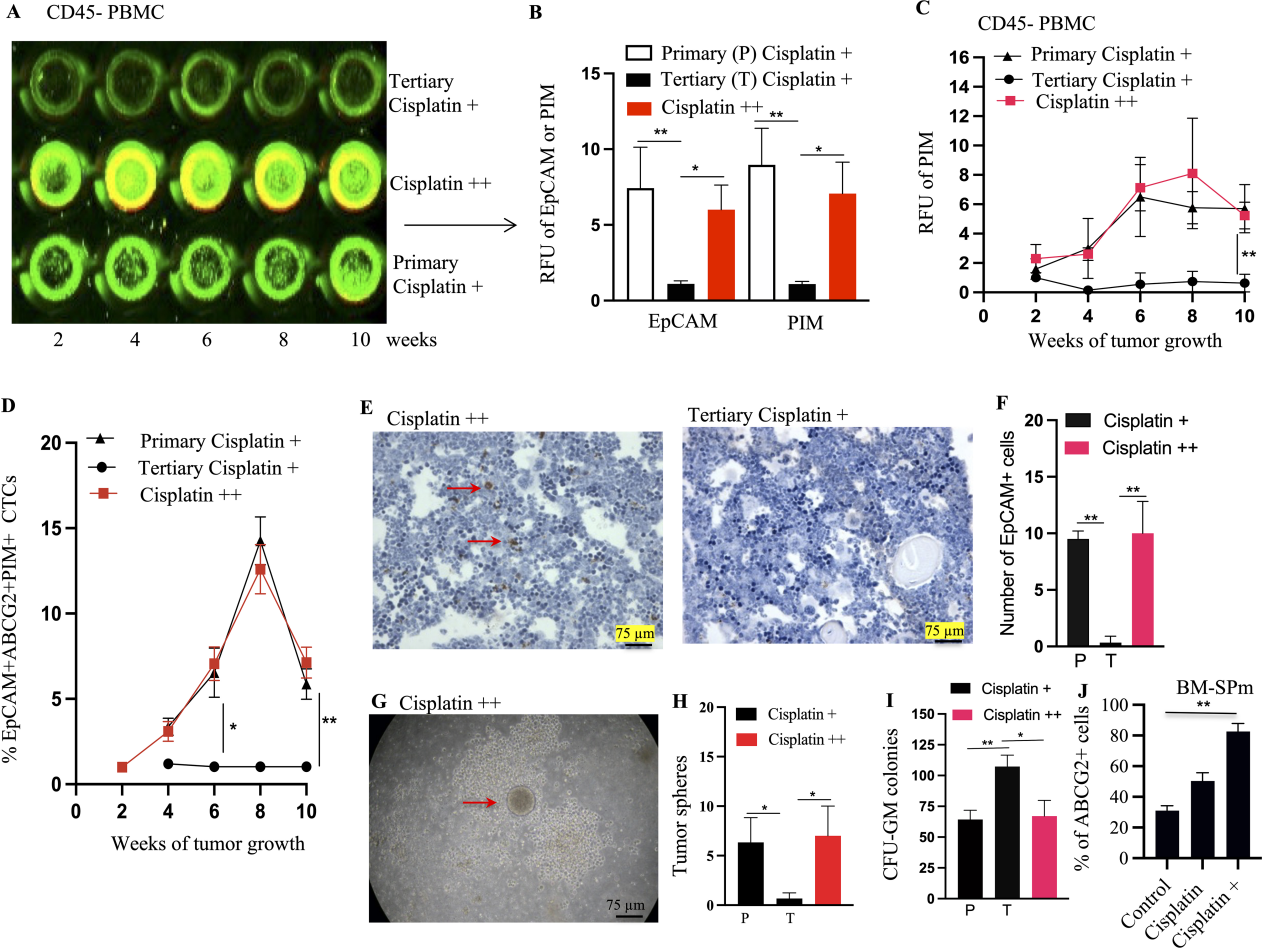
Mobilization of EpCAM+/ABCG2+ cells (enriched in TSD+ CSCs) from cisplatin ++ xenograft. **(A, B)** In cell Western of EpCAM (green) and ABCG2 (yellow) proteins in CD45 negative PBMCs isolated from cisplatin ++ xenograft (week 8). These cells likely represent TSD+ CTCs. RFU denotes relative fluorescence unit. **(C)** In Cell ELISA reveals a progressive increase in pimonidazole levels in the CD45 negative PBMC. **(D)** Progressive increase in the percentage of EpCAM+/ABCG2+/PIM+ cells in the CTCs of primary cisplatin + and Cisplatin ++ tumors, suggesting rising TSD+ CTCs. PBMC derived CD45 negative cells were expanded in the injured conditioned media for a week to obtain tumorospheres, and then, the EpCAM+/ABCG2+/PIM+ cells cell percentage was quantified in the dissociated tumorospheres by flow cytometry. **(E, F)** Immunohistochemistry detects EpCAM+ cells (red arrows) in the bone marrow (BM) of tertiary cisplatin+ and cisplatin ++ mice [quantified in **(F)**]. **(G)** A tumorosphere assay image shows a tumorosphere embedded within a CFU-GM colony in BM derived mononuclear cells cultured in methylcellulose media with GM-CSF (quantified in **H&I**). Since directly isolating EpCAM+ CTCs can be challenging, we used CD45 negative cells from PBMC of xenograft-bearing mice to enrich EpCAM+/ABCG2+ CTCs. **(J)** The BM spheroid derived EpCAM+/ABCG2+ cells were subjected to TSD phenotype assays described in **Figure 6G**. The R-CSCs in the BM spheroids show similar number of ABCG2 cells as the primary tumor data 6G indicating reawakening. Data represent ± SEM, three independent experiments, One Way ANOVA is for **(B**, **H**, **I)**. Student’s t-test is for **(C**, **D**, **F**, **J)**. *p<0.05, **p<0.01.

**Figure 10:**
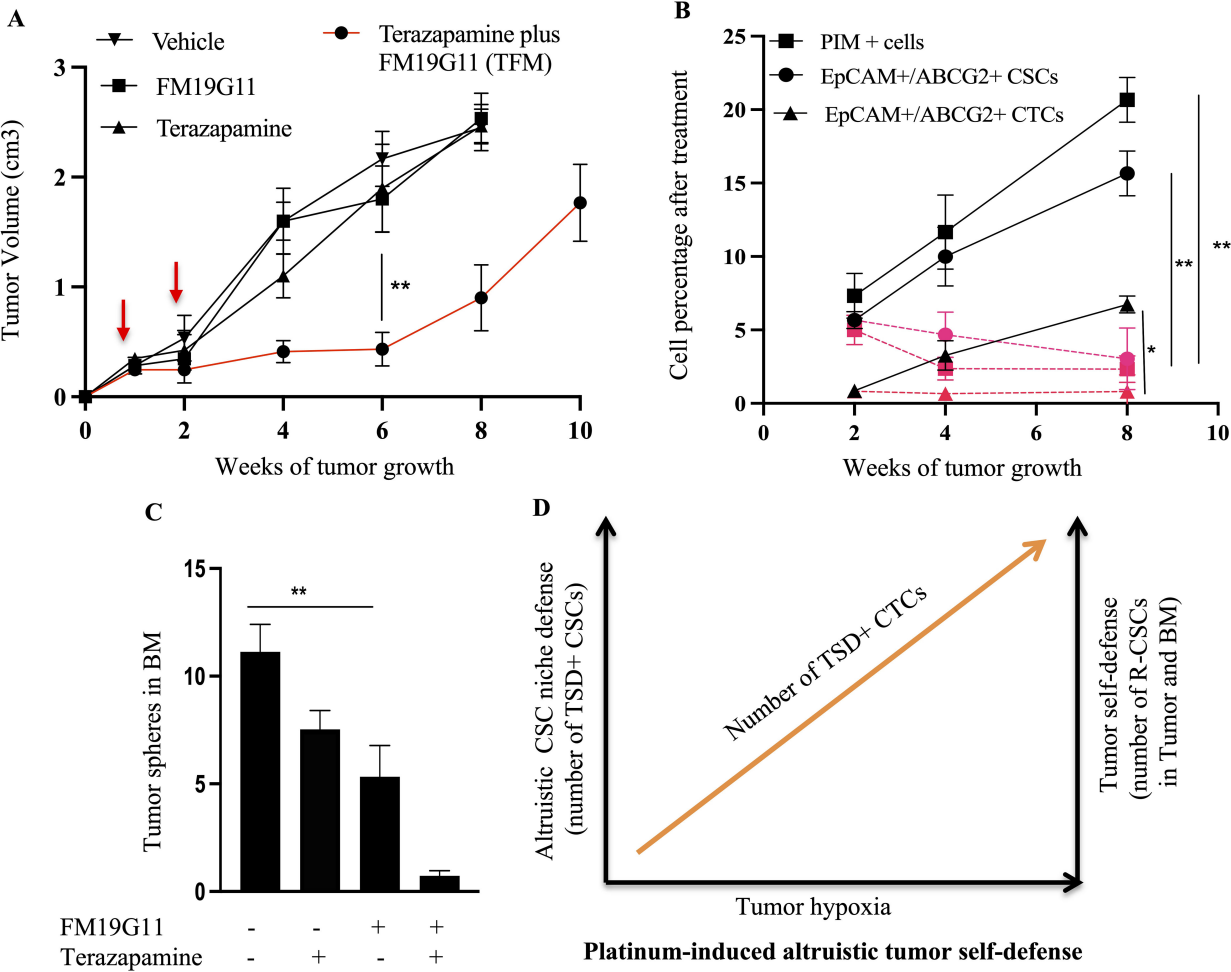
Effect of terazapamine and FM19G11 (TFM) on tumor growth, CSCs, CTCs, and bone marrow (BM). **(A)** Treatment with TFM (combination of Terazapamine and FM19G11) significantly reduces tumor growth in cisplatin++ xenograft mice. **(B)** Cisplatin++ xenografts exhibit increase of CSC niche defense (PIM+ cells, TSD+CSCs, TSD+CTCs, R-CSCs), which is decreased after TFM treatment as shown in red color. Please note that R-CSC is enriched in the EpCAM+/ABCG2+ CSCs and CTCs. **(C)** TFM significantly reduces EpCAM+ cell homing to the bone marrow in cisplatin++ xenograft mice. **(D)** This schematic proposes a model where platinum exposure triggers dormant CSCs to transform into TSD+ CSCs, and R-CSCs, as a part of altruistic tumor self defense. These TSD+ CSCs might exhibit altruistic behavior, supporting the niche as well as R-CSCs for future tumor growth. The R-CSCs re-enter dormancy while retaining a "memory" of past stress that allows them to reawaken upon encountering similar stress (like cisplatin) in the future. This memory-driven reactivation of R-CSCs and the associated altruism of the TSD+ phenotype contributes to chemoresistance. **(A–C)** Data represents ± SEM, n=4 independent experiments, student t test, ** p<0.01.

Our findings suggest that TSD+ CSCs, a subpopulation of EpCAM+/ABCG2+ CSCs, might engage in a form of self-sacrifice to protect the R-CSC, a pool of dormant CSC population capable of reawakening upon encountering future cisplatin-induced stress and resuming tumor growth. This altruistic behavior of TSD+ CSCs could ensure tumor survival and reseeding during future cisplatin-induced stress by preserving the R-CSC pool. Additionally, TSD+ CSCs might altruistically contribute to the dissemination of R-CSCs to metastatic niches through the mobilization of CTCs in the hypoxic niches of the tumor. Supporting this hypothesis, EpCAM+/ABCG2+ CTCs were only detected in the primary transplant (cisplatin+) and during R-CSC reawakening (cisplatin++; **Figures 9A–C**). Notably, these CTCs were absent in the secondary or tertiary transplants. Interestingly, the cisplatin++ group mirrored the cisplatin+ group by exhibiting an increase in EpCAM+/ABCG2+/pimonidazole+ CTCs between the 2nd and 8th week of tumor growth (**Figure 9D**). This rise in CTCs displayed a strong association with tumor hypoxia (*r* ^2^ = 0.76, *p* < 0.03) (**Supplementary Figure 9**). Furthermore, tumor spheroids and EpCAM+ cells were only detected in the bone marrow (BM) of mice with the cisplatin+ and cisplatin++ group (**Figures 9E–I**), similar to the pattern observed for CTCs. Moreover, EpCAM+/ABCG2+ cells obtained from the BM tumor spheroids (Cisplatin ++ group), when injected to mice, showed induction of TSD phenotype (**Figure 9J**) similar to the parental EpCAM+/ABCG2+ cells (**Figure 6G**). This result indicates that the EpCAM+/ABCG2+ cells residing in BM contain R-CSCs. Finally, therapeutic targeting of hypoxia through treatment with terazapamine and FM19G11 (a small molecule inhibitor of HIF-1α and HIF-2α) resulted in a marked reduction of tumor growth and a corresponding decrease in TSD+ CSCs and CTCs and the presence of R-CSCs within the BM (**Figures 10A–C**).

These findings suggest a potential link between tumor hypoxia, TSD+ CSCs’ mobilization, and an altruistic CSC niche defense mechanism. In this mechanism, a subset of EpCAM+/ABCG2+ cells (TSD+ CSCs) behave altruistically to protect the dormant R-CSCs, ultimately enhancing the overall fitness of the tumor (**Figure 10D**). Our results also indicate that the EpCAM+/ABCG2+ cell population is heterogenous, containing two distinct sub-populations: the TSD+ CSCs capable of reprogramming to a more aggressive phenotype and the R-CSCs that retain a stress memory.

## Discussion

This study explores a potential altruistic mechanism in CSCs of HNSCC. We explored a putative altruistic CSC niche defense as the underlying cause of the periodic burst of CTCs. In HNSCC patients receiving platinum therapy (*n* = 14), we identified a novel TSD+ CSC and TSD+ CTC phenotype characterized by the transient expansion and expression of TSD+ phenotype in EpCAM+/ABCG2+ CSCs. Importantly, we have established a pre-clinical model of cisplatin-induced expansion of TSD+ CSCs and TSD+ CTCs in NOD/SCID mice. Treatment with cisplatin reprogrammed dormant CSCs to TSD+ CSCs in the hypoxic niche. The expansion of TSD+ CSCs coincided with a temporary burst of TSD+ CTCs, mimicking clinical observations. These pre-clinical and limited clinical findings suggest a link between TSD+ CSCs and cisplatin resistance. Further research is needed to understand this novel altruistic CSC phenotype and its role in cancer progression.

Our findings in the pre-clinical model suggest that cisplatin treatment might induce a stress response, leading to hypoxia and activation of the CSC niche defense mechanism. This mechanism appears to involve the reprogramming of quiescent EpCAM+/ABCG2+ CSCs to the TSD phenotype, characterized by transient expansion (rapid proliferation under hypoxic conditions), cytoprotective factor secretion (secretion of factors like glutathione (GSH), potentially protecting neighboring tumor cells from cisplatin-induced toxicity), and altruistic apoptosis (a transient state that undergoes spontaneous apoptosis *in vitro* and disappears during serial transplantation *in vivo*, suggesting a potential self-sacrificing mechanism for the benefit of the tumor self-identity). Furthermore, conditioned media obtained from these self-sacrificing cells protected the neighboring EpCAM+/ABCG2- cells grown under starvation or cisplatin. This intriguing observation suggests the secretion of protective factors by these cells while maintaining stemness. However, conditioned media experiments cannot definitively prove the mechanism by which these factors exert their protective effect. Further studies are needed to isolate and characterize the specific factors involved and elucidate the signaling pathways underlying this potential altruistic behavior of TSD+ EpCAM+/ABCG2+ CSCs.

The use of injured conditioned media (ICM) to expand TSD+ CSCs raises the possibility that the MSCs used in its preparation might be involved in CSC niche defense. The tumor-exposed MSCs could be reprogrammed into a supportive state, akin to “altruistic mesenchymal stem cells” (A-MSCs). We observed the presence of cells with characteristics suggestive of A-MSCs when exposing MSCs to conditioned media obtained from A-CSCs (29). However, further investigation is required to definitively characterize these cells and their functional role in supporting TSD+ CSCs during platinum therapy.

Our data suggest a link between EpCAM+/ABCG2+ CSCs residing in the hypoxic niche and TSD+ CTCs. We observed a correlation between the presence of these CSCs in primary tumors and the detection of TSD+ CTCs in the blood. Furthermore, functional characteristics of CSCs isolated from patients with TSD+ CTCs displayed properties associated with the TSD phenotype. EpCAM+/ABCG2+ CTCs isolated from these patients also showed pimonidazole staining, suggesting their origin from the hypoxic niche. However, further investigation is needed to definitively confirm the direct shedding of CSCs into the bloodstream.

Interestingly, a recent study suggests that some cancer cells exposed to hypoxia develop a “hypoxic memory” that persists even after reoxygenation (30). Future research is needed to investigate whether the altruistic CSC niche defense contributes to the formation of this hypoxic memory by protecting the R-CSCs. We proposed that the preservation of R-CSCs indicates the preservation of tumor self-defense or tumor self-identity.

We propose the activation of the MYC-HIF-2α stemness pathway as a potential mechanism underlying the TSD phenotype following cisplatin treatment. Our *in vivo* results indicate that the inhibition of HIFs by FM19G11, as well as its downstream effectors of the pathway, VEGF/VEGFR1 autocrine loop (31), and EGF/EGFR autocrine loop markedly reduced tumor growth as well as the expansion of the EpCAM+/ABCG2+ cells. Previously, we reported that the VEGF/VEGFR1 pathway was involved in the cisplatin-induced activation of the tumor stemness switch (TSD phenotype) (9). The current findings further our ongoing research in the mechanism of platinum-induced tumor stemness. Moreover, our previous findings on the MYC-HIF-2alpha stemness pathway (20) are supported by the evidences from other studies on human cancer (32, 33).

While the pre-clinical model provided valuable insights, it has limitations in addressing specific questions regarding the true nature of TSD+ CSC behavior—altruistic (self-sacrifice) or selfish (self-preservation). Our investigation into the behavior of TSD+ CTCs in the bone marrow niche suggests a more nuanced scenario. These cells did not self-colonize but created a pre-metastatic niche for a distinct population—the R-CSCs (reawakening cancer stem cells)—as evidenced by our bone marrow localization of EpCAM+ cells. This highlights the potential existence of memory within the CSC population, aligning with the “altruistic CSC niche defense” hypothesis. However, further research is needed to definitively confirm or refute the altruistic nature of TSD+ CSCs.

CTCs have been identified in HNSCC, but the source is not yet clear. Further research is crucial to decipher the true nature of TSD+ CSC behavior (altruistic vs. selfish) and these cells as the potential source of CTCs in HNSCC. The inducible TSD phenotype in CSCs and CTCs has significant clinical implications, including the development of novel therapeutic strategies such as BCG-mediated immunotherapy and biomarker development to monitor platinum therapy response.

In conclusion, this study sheds light on the TSD+ phenotype in CSCs, potentially linked to an altruistic self-defense mechanism within tumors. Our findings suggest that hypoxia induced by cisplatin treatment reprograms EpCAM+/ABCG2+ CSCs to TSD+ cells exhibiting transient expansion, cytoprotective factor secretion, and altruistic apoptosis. While the origin of TSD+ CTCs and the truly “altruistic” nature of TSD+ CSCs require further investigation, the potential subpopulations with varying levels of self-preservation and niche protection highlight the complexity of CSC biology. Understanding the interplay between hypoxia, CTC mobilization, and the CSC niche defense mechanism is crucial to develop effective cancer therapies. The inducible TSD phenotype holds promise for improved therapy prediction based on the presence of TSD+ CSCs, development of novel therapeutic strategies targeting the TSD+ CSC niche or the MYC-HIF-2α stemness pathway, and the use of TSD+ CTCs as a biomarker for treatment response in HNSCC. Future research will focus on deciphering the true nature of TSD+ CSC behavior and unlocking the full potential of targeting this CSC phenotype for improved patient outcomes.

## Data Availability

The original contributions presented in the study are included in the article/**Supplementary Material**, further inquiries can be directed to the corresponding author/s.
