# Supplementary material for: Hypoxia-driven mobilization of altruistic cancer stem cells in platinum-treated head and neck cancer
Source: Front Immunol. 2025 Feb 3;15:1336882. doi: 10.3389/fimmu.2024.1336882 (PMC11830676; doi:10.3389/fimmu.2024.1336882)
Supplement: Supplementary file 1 [file SupplementaryFile1.pdf]

Supplementary Figure 1: Expansion of TSD+ CTCs in the "injured conditioned media' (ICM).

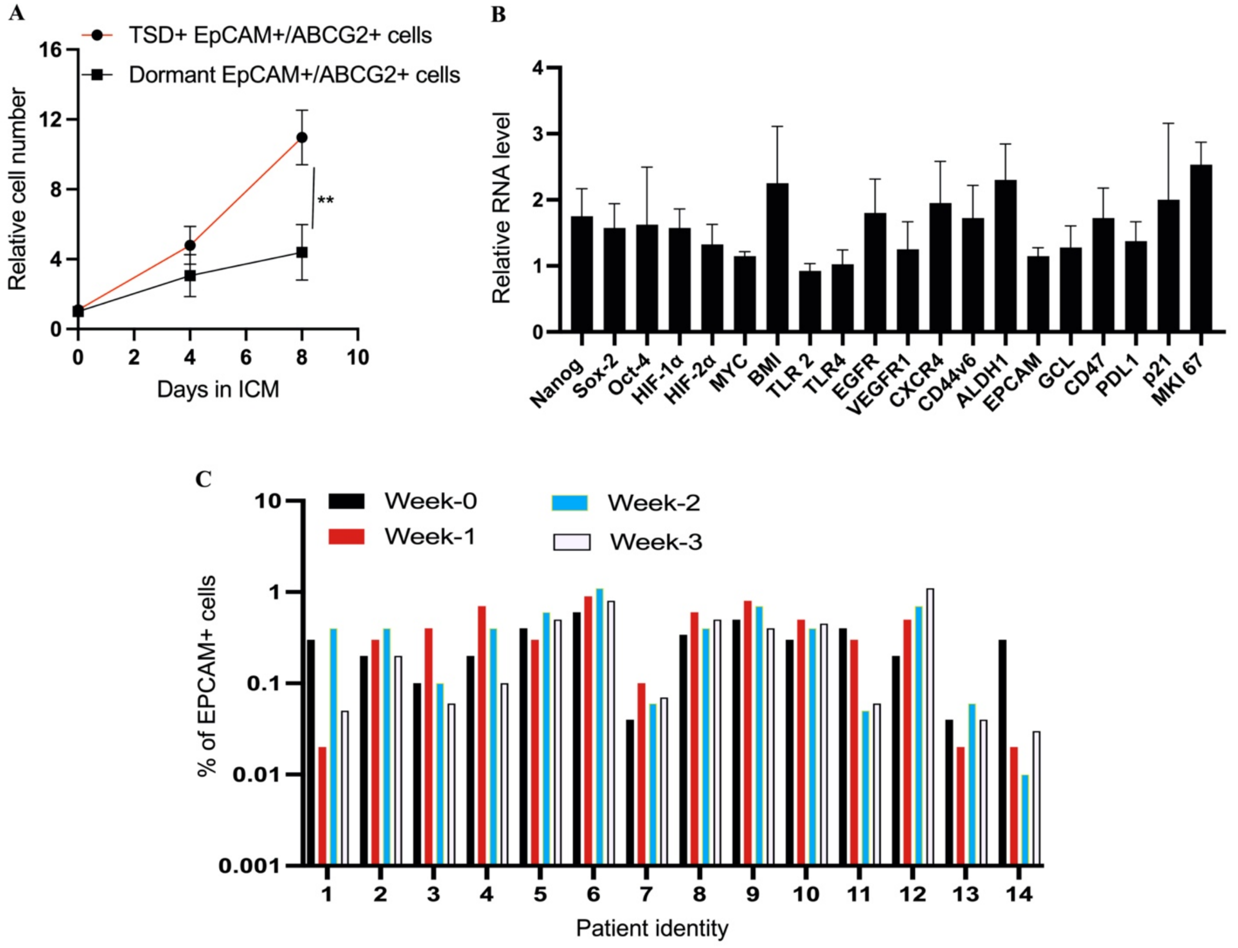

A.The Injured conditioned media (ICM) does not induce the TSD+ phenotype to EpCAM+/ ABCG2+ cells. However, it can expand EpCAM+/ABCG2+ cells with the TSD phenotype. The TSD+ EpCAM+/ABCG2+ cells were obtained from the intermittent hypoxia exposed SCC-25 cell line as previously described (12). The dormant EpCAM+/ABCG2+ cells were flow cytometry sorted from growing SCC-25 cell culture. The SCC-25 cells were grown under 2% O2 in DMEM/F12 media with 10% FBS, and hydrocortisone for a week to collect the slow growing EpCAM+/ABCG2+ cells. Data represent ± SEM; student t test ;** $p<0.01$. **B.** The real time PCR gene expression of the dormant EpCAM+/ABCG2+ cells grown in the ICM for a week. The gene expression data was compared with the dormant EpCAM+/ABCG2+ cells. **C.** Flow cytometry analysis of PBMC of platinum-treated HNSCC subjects (Supplementary table 1).

Supplementary Figure 2: Altruistic behavior of the EpCAM+/ABCG2+ cells obtained from the CTC-positive group of patients

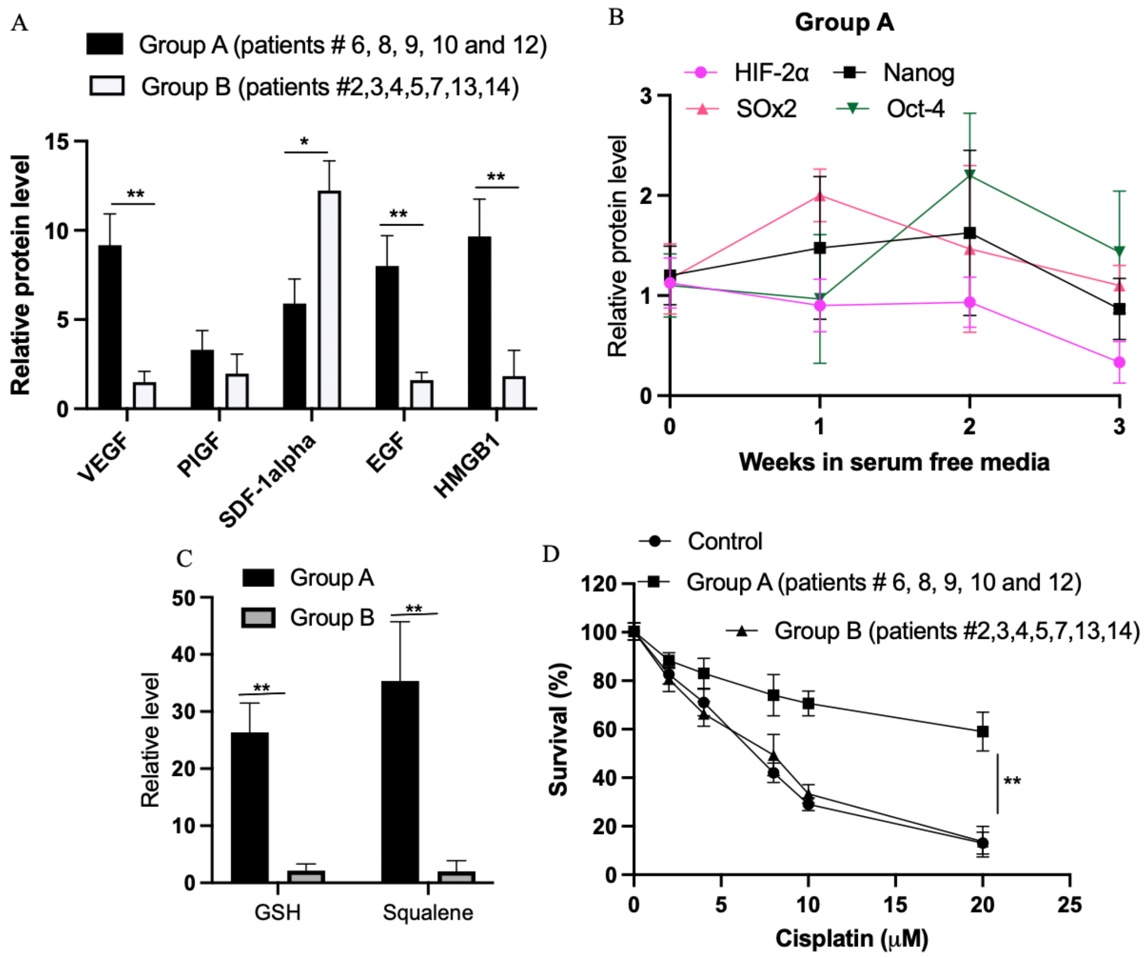

A. Growth factors and HMGB1 secreted by EpCAM+/ABCG2+ cells grown in the serum free media with 2% Oxygen for two weeks, as measured by ELISA. **B**. The EpCAM+/ABCG2+ cells of group A patients maintain self-sufficiency (stemness) when grown in the serum-free media with 2% Oxygen for three weeks. **C.** GSH (glutathione) and Squalene secretion by the EpCAM+/ABCG2+ cells (group A) grown in the serum free media with 2% Oxygen for two weeks. GSH and squalene were measured as previously described (23). **D.** The cytoprotection of EpCAM+/ABCG2- cells from cisplatin-induced toxicity when treated with the conditioned media (CM) of the EpCAM+/ABCG2+ cells of Group A versus Group B grown for two weeks. The CM was collected from 1x10^3 cells grown in 500 μl serum free media for 48 hours. Control group: the EpCAM+/ABCG2- cells were grown in the "injured conditioned media" with cisplatin for 72 hours. Cell survival was measured by the trypan blue assay. data represent ± SEM; A: ONE WAY ANOVA, C-D: student t test .*p<0.05, ** p<0.01, *** p<0.001

Supplementary Figure 3: Primary tumors used for the patient-derived xenograft study.

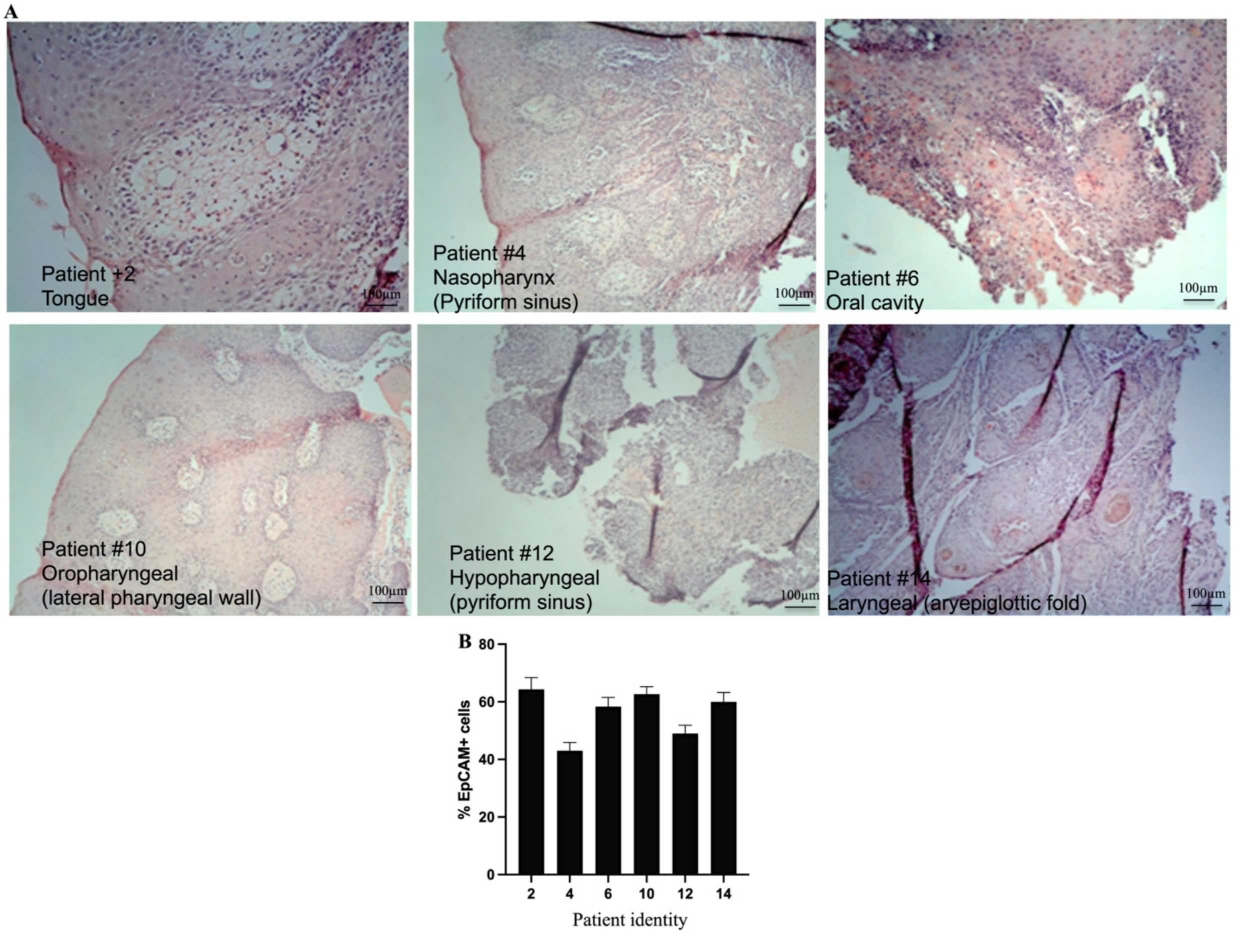

A. The H&E section of the patient-derived tumors. B. The percentage of EpCAM+ cells in the tumors as measured by flow cytometry. The surgical specimens were collected (~2 gm total tissues from 3-4 different zones of the resected tumor). Each zone was dissociated separately to assess the tumor hypoxia (pimonidazole positive cells), and the percentage of the EpCAM+, as well as EpCAM+/ABCG2+ cells (given in the main text, Figures 3D-E) were assessed by flow cytometry. In **B**, the mean values of the EpCAM+ cells are shown.

Supplementary Figure 4: Analysis of Tumor Hypoxia, and TSD+ Phenotype in patient-derived primary tumors.

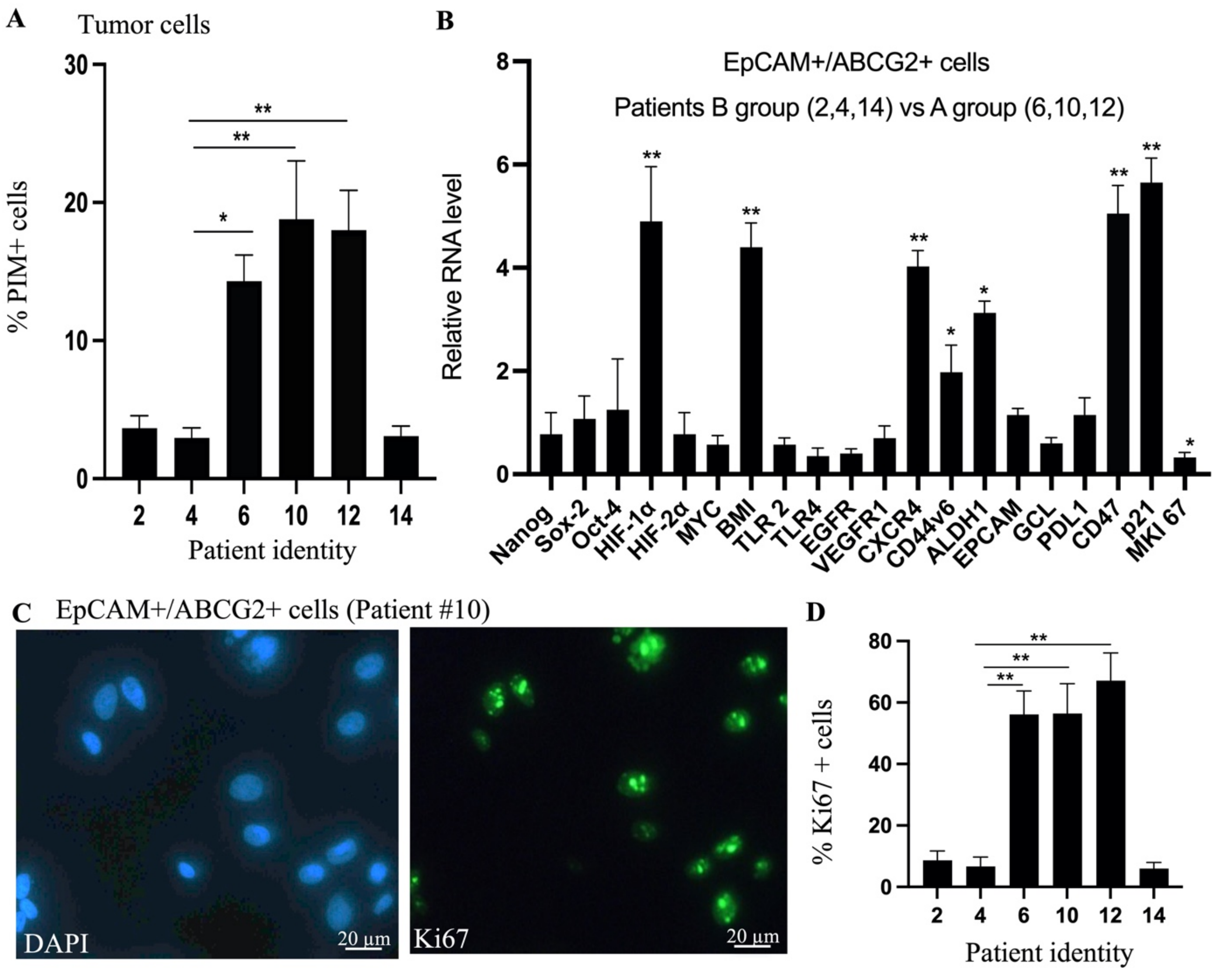

A: Pimonidazole binding was measured by flow cytometry in the bulk tumor cells from all patients to assess overall tumor hypoxia. Mean values are shown. **B:** qPCR data showing the downregulation of genes associated with the MYC-HIF-2alpha stemness pathway in the EpCAM+/ABCG2+ cells of patients B vs A. Note that the expression of a dormancy-related gene CD47 is highly expressed in the B group. CD47 is the dormancy and immunosuppressive gene. **C:** An immunofluorescence photomicrograph shows Ki67 staining (green: Ki-67; blue: Dapi) in TSD+ EpCAM+/ABCG2+ cells from patient 10. The accompanying histogram quantifies Ki67-positive cells per 100 Dapi-stained cells. **D.** Quantification of the immunofluorescence data shown in C. Data are represented as mean ± SEM; One Way ANOVA, * $p<0.05$, ** $p<0.01$, *** $p<0.001$.

Supplementary Figure 5: Analysis of Cytoprotective Effects exerted by primary tumor derived TSD+ CSCs grown in serum-free media under 2% Oxygen.

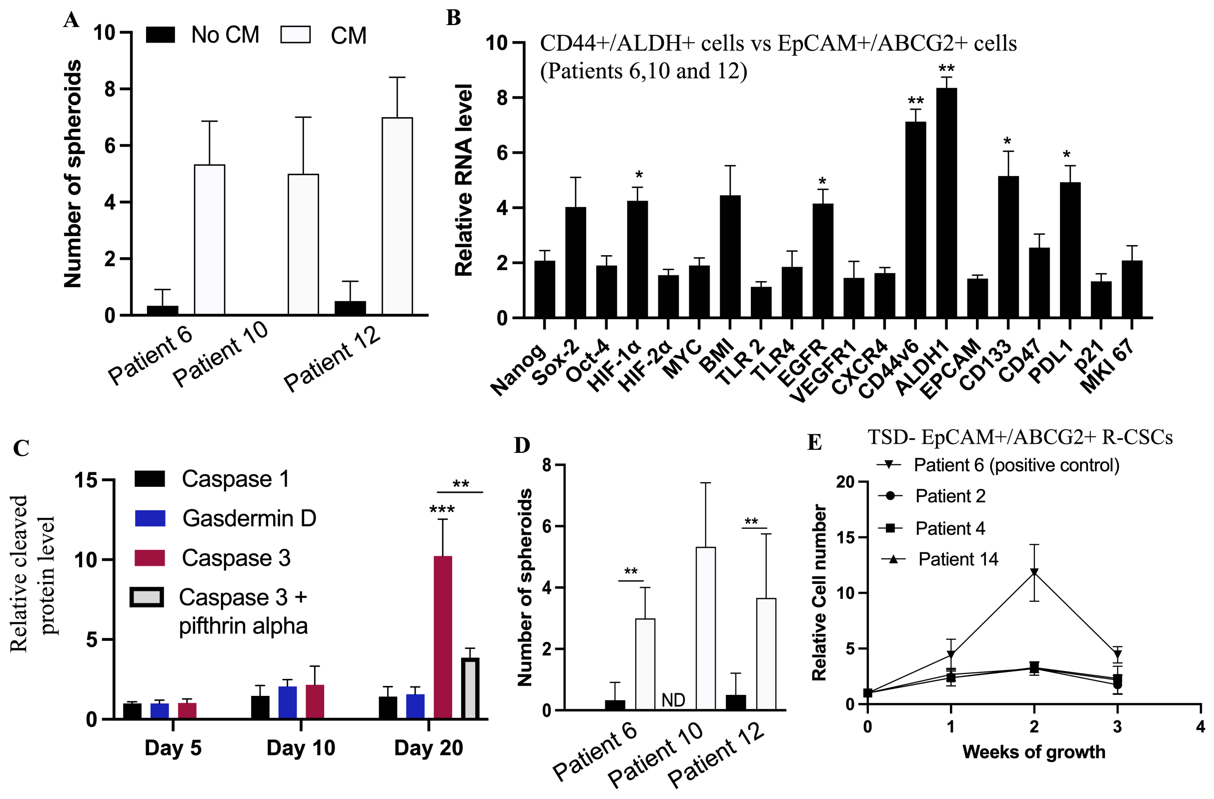

A. A spheroid culture assay shows conditioned media from proliferating TSD+ CSCs (day-10 grown in serum-free media under 2% Oxygen, details in Figure 4E legend, main text) protects EpCAM+/ABCG2- cells from serum-starvation induced cytotoxicity (day-16 growth in serum-free media under 2% Oxygen). **B**: qPCR Analysis of TSD+ Gene Expression in CD44+/ALDH+ cells grown in injured conditioned media under 2% Oxygen. The gene expression was compared with the EpCAM+/ABCG2- cells grown under similar conditions. The data from the patients 6, 10 and 12 were combined. **C:** Activation of cell death pathways in day-21 EpCAM+/ABCG2+ CSCs grown in serum-free media under 2% oxygen. The relative protein levels of cleaved caspase-1, Gasdermin D, and cleaved caspase 3 were measured by ELISA, and the data from patients 6, 10, and 12 were combined for statistical analysis. To investigate the role of p53 in cell death, a separate group of cells was treated with pififthrin alpha (10 um for four days, starting at day 16), a p53 inhibitor. The results showed an increase in cleaved caspase-3 level, inhibited by pifithrin alpha, indicating a potential p53-dependent pathway for caspase-3 activation in these cells. **D:** Spheroid culture assay examines paracrine protection of EpCAM+/ABCG2- cells by dying TSD+ CSCs. Here, the examination described in Panel A was repeated with the conditioned media collected from day-21 EpCAM+/ABCG2+ CSCs (patient #6 and 10) grown in serum-free media under 2% Oxygen. **E:** The dormant behavior of TSD negative EpCAM+/

ABCG2+ CSCs (Patient 2, 4 and 14) grown under serum free media with 2% Oxygen. These dormant TSD negative EpCAM+/ABCG2- cells are enriched in R-CSCs as explained in the main text; Figure 4G result. As a comparison, TSD+ EpCAM+/ABCG2+ CSCs from patient 6 were used. Data are represented as mean ± SEM. Statistical significance was determined using One Way ANOVA is done for A, B, C, E. *p < 0.05, **p < 0.01.

Supplementary Figure 6: Cisplatin-stress response and dormancy in SCC-25 derived migratory SP cells, and EpCAM+/ABCG2+ cells.

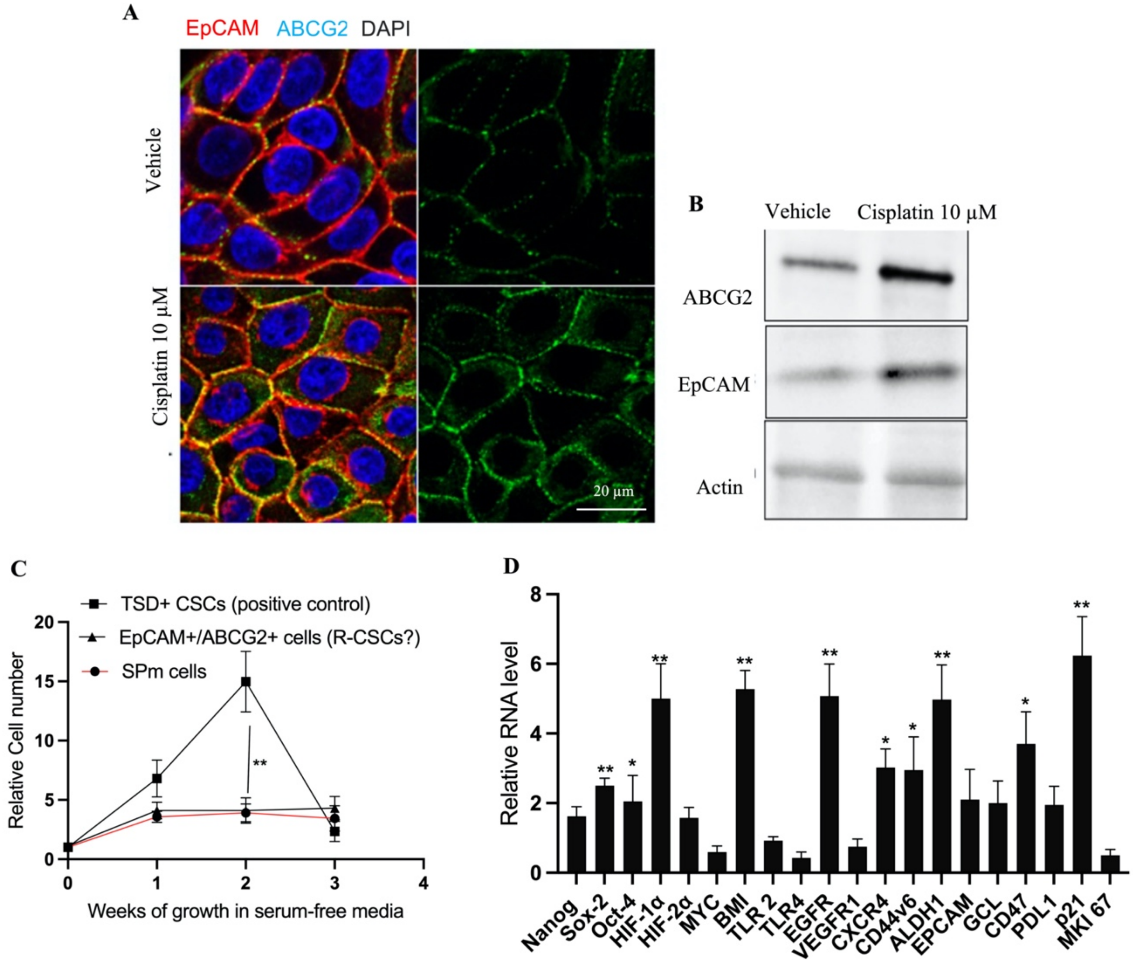

A & B. Cisplatin treatment (10 uM for four days under serum free media with 2% Oxygen) of SCC-25 cells increased EpCAM & ABCG2 in migratory SP (SPm) cells. Immunofluorescence confirmed higher EpCAM & ABCG2 protein on SPm surface. Western blot showed similar protein increase after a week of growth in the injured conditioned media under 2% Oxygen. **C:** EpCAM+/ABCG2+ cells are enriched in R-CSCs as explained in the main text; Figure 5 result. EpCAM+/ABCG2+ cells and SPm cells obtained from cisplatin treated SCC-25 cells lacked the

ability to stress-induced expansion, when grown in serum free media with 2% Oxygen. The patient-6 derived TSD+ EpCAM+/ABCG2+ CSCs was used as a positive control for the TSD+ phenotype behavior under serum-free conditions. **D:** EpCAM+/ABCG2+ cells obtained from cisplatin treated SCC-25 cells lacked TSD+ gene expression but showed high expression of dormancy genes; HIF-1alpha, EGFR, CXCR4 and p21. This was also observed in patient 2,4 and 14 derived dormant EpCAM+/ABCG2+ CSCs. The qPCR compared TSD+ genes in EpCAM+/ABCG2+ cells from cisplatin vs. vehicle treatment grown in the injured conditioned media with 2% Oxygen for two weeks. Data are represented as mean ± SEM. Statistical significance was determined using a Student's t-test for C, and One WAY ANOVA for D. *p < 0.05, **p < 0.01.

Supplementary Figure 7: TSD+ Phenotype and altruistic behavior of Xenograft-Derived (cisplatin + group) CTC

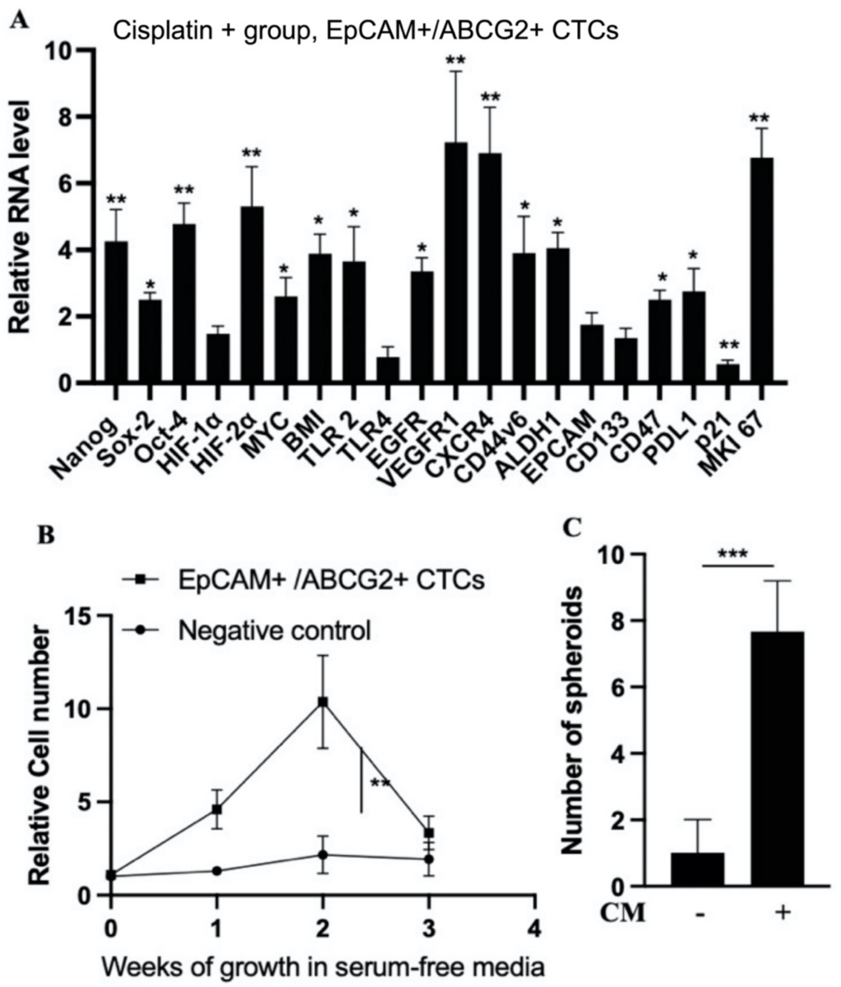

A: TSD+ phenotype genes were analyzed in EpCAM+/ABCG2+ CTCs isolated from mice treated with cisplatin (8-week xenografts, cisplatin+ group). PBMC enriched in CD45 negative fraction was first expanded in "injured conditioned media" for a week, EpCAM+/ABCG2+ cells

were isolated by immunomagnetic sorting. Their gene expression was compared to EpCAM+/ ABCG2- cells obtained from the same culture. **B**. **Self-sufficiency:** CTC spheroid derived EpCAM+/ABCG2+ cells were grown in serum-free media under 2% Oxygen to assess their transient ability to expand under nutrient-depleted conditions. The in vitro post-cisplatin treated EpCAM+/ABCG2+ cells (Supplementary Figure 6C) served as negative control. **C. Altruistic Behavior:** Conditioned media was collected from days 18-21 EpCAM+/ABCG2+ CTC (panel B). This conditioned media was then tested for its ability to cytoprotect EpCAM+/ABCG2- CTCs (obtained from the spheroids; panel A) grown under serum free media and 2% Oxygen. Data are represented as mean ± SEM. Statistical significance was determined using ONE WAY ANOVA for A, and Student's t-test for B. *p < 0.05, **p < 0.01.

Supplementary Figure 8: TSD+ phenotype exhibits the activation of MYC-HIF-2alpha stemness pathway

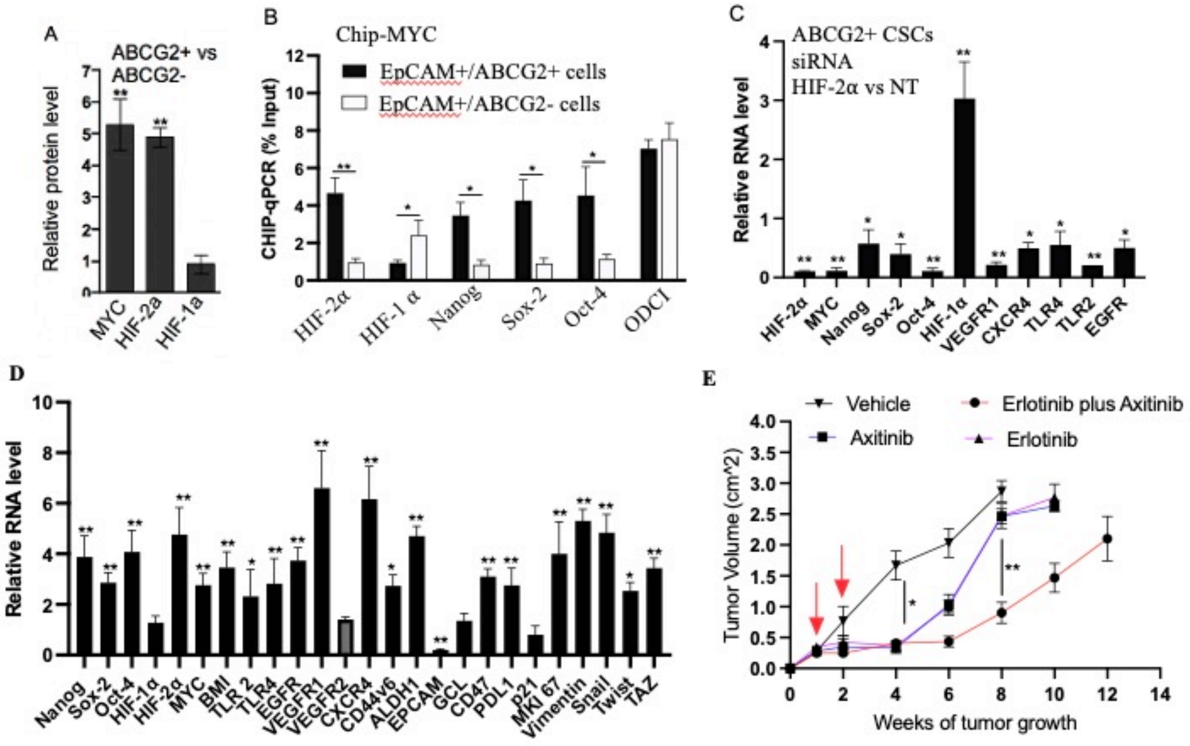

A &B: The EpCAM+/ABCG2+ cells obtained from the Cisplatin+ xenografts (8 weeks) exhibit the activation of MYC- HIF-2α stemness pathway. Panel A shows protein levels measured by ELISA, and Panel B shows MYC binding to HIF-2α, Nanog, Sox-2 and Oct-4 promoters. The qPCR analysis of ChiP-MYC in EpCAM+/ABCG2+ and ABCG2- cells were performed at indicated promoters. The method of Chip-qPCR is described previously (19). ODCI, a MYC target gene served as positive control. The IgG panel represent matched isotype. Data is

quantified as percentage of input DNA (N=3 independent samples; Student t test). **C:** qPCR data indicates the genes levels in HIF-2alpha silenced EpCAM+/ABCG2+ cells. HIF-2alpha silencing was achieved by accell siRNA (19). NT: non-targeting. **D**: qPCR gene expression analysis of EpCAM-/ABCG2+ cells from cisplatin + xenografts (8 weeks; details in Fig 5F-G). These cells may have undergone EMT due to EpCAM loss. TSD+ phenotype and EMT related genes (Vimentin, Snail, Twist, Trail) were analyzed. **E:** The resistance of SCC-25 derived cisplatin + xenograft growth to Erlotinib (80 mg/kg daily for two weeks; i.p.) and Axitinib (25 mg/kg daily for two weeks; i.p.) treatment. These two drugs were given along with cisplatin (10mg/kg once weekly for two weeks; red arrows). Noted that the combination treatment significantly reduced tumor growth at 8th weeks. Each group contained n=5 mice, and the experiments were repeated thrice.

Supplementary Figure 9: The rise in tumor hypoxia is associated with the increase in CTC

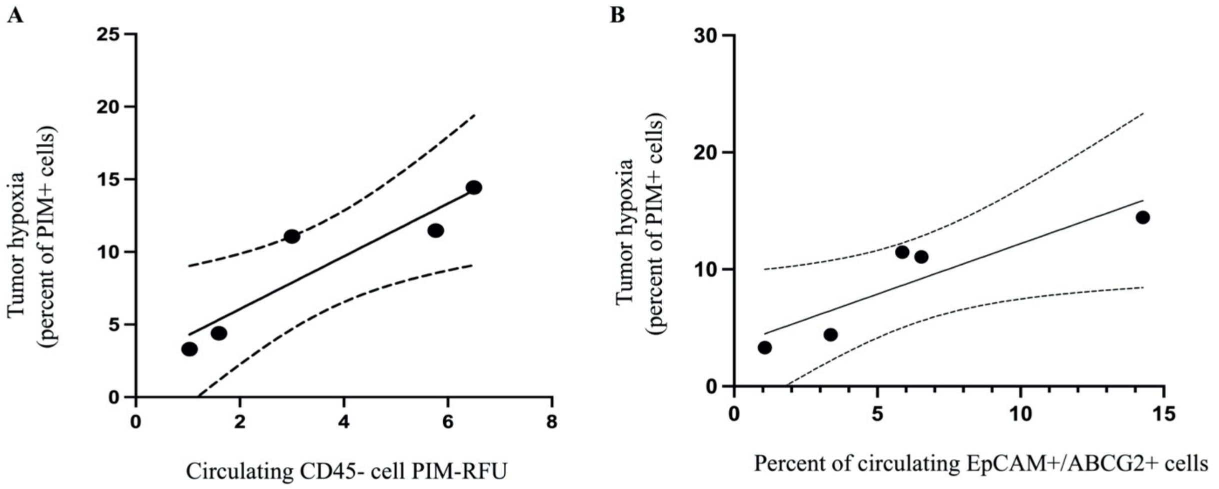

A. data of Figure 8E and Figure 9C were subjected to correlation analysis; r2= 0.79, p<0.04.
B. data of Figure 8E and Figure 9D were subjected correlation analysis; r2 =0.76, p<0.03.

Using the correlation statistic of Prism, an association between the tumor hypoxia (Figure 8E) data and the PIM-RFU levels of circulating CD45- cells (Figure 9B), or the PIM+ CTC (Figure 9D), where the number of CTCs was obtained by an indirect method of expanding circulating CD45- cells in vitro tumorosphere assay (as described in the Figure 9D legend). The linear regression was performed to obtain the line, and the 95% confidence interval for the slope.

References:
12. Bhuyan S, Pal B, Pathak L, Saikia PJ, Mitra S, Gayan S, et al. Targeting hypoxia- induced tumor stemness by activating pathogen-induced stem cell niche defense. *Front Immunol*. (2022) 13:933329. doi: 10.3389/fimmu.2022.933329.
19. Das B, Pal B, Bhuyan R, Li H, Sarma A, Gayan S, et al. MYC Regulates the HIF2a Stemness Pathway via Nanog and Sox2 to Maintain Self-Renewal in Cancer Stem Cells versus Non-Stem Cancer Cells. *Cancer Res*. (2019) 79:4015–25. doi: 10.1158/0008- 5472.CAN-18-2847

23. Das B, Antoon R, Tsuchida R, Lot! S, Morozova O, Farhat W, et al. Squalene selectively protects mouse bone marrow progenitors against cisplatin and carboplatin- induced cytotoxicity in vivo without protecting tumor growth. *Neoplasia*. (2008) 10:1105–19. doi: 10.1593/ neo.08466.

List of Antibodies used:

Protein name	Antibody	ELISA kit
HIF-2α	AB199; Abcam (Flow cytometry)	MBS702348 Mybiosource, CA
HIF-1α		DYC1935-2, R&D
Oct-4		#9539, Cell Signaling
Nanog		Ab236720, Abcam
Sox2		#245707, Abcam
Myc		KA3254, Abnova, CA, USA
ABCG2	AB3380; Abcam (WB,IF, Flow cytometry)	MBS703358 Mybiosource, CA
Cleaved caspase-3		DYC835-2, R&D
Cleaved caspase-1	#A1004 rabbit polyclonal (Biovision, Milpitas, CA). #sc-56036 mouse monoclonal (Santacruz)	In house Sandwich ELISA
Gasdermin D	#36425 rabbit polyclonal (Cell Signaling Technology) and #H00079792-M01 (Abnova).	In house Sandwich ELISA

Protein name	Antibody	ELISA kit
VEGF		#DVE00, R&D Systems
PIGF		#DPG00, R&D Systems
SDF 1alpha		#DSA00, R&D Systems
HMGB1		#NBP2-62766, Novus Biologics
EGF		#KHG0061, Invitrogen
EpCAM	#14-9326-82, Invitrogen (WB,IF, Flow cytometry)	
β-Actin	#4967, Cell Signaling Technology (WB)	
Pimonidazole	Rabbit anti pimonidazole antibody, Hypoxyprobe store, NPI inc (Flow cytometry, ELISA)	In house direct ELISA
Ki67	#9129, Cell Signaling Technology, (IF)	
