# Supplementary material for: Hypoxia-driven mobilization of altruistic cancer stem cells in platinum-treated head and neck cancer
Source: Front Immunol. 2025 Feb 3;15:1336882. doi: 10.3389/fimmu.2024.1336882 (PMC11830676; doi:10.3389/fimmu.2024.1336882)
Supplement: Supplementary file 2 [file Table1.pdf]

Supplementary table 1: The table is presenting the patients information for 14 subjects included in the study.

Clinical Characteristics	Cases, n (%)
All cases	14 (100)
**Age**	
35-49<br>50-65 years	5 (35%)<br>9 (64.28%)
**Sex**	
Male<br>Female	8 (57%)<br>6 (42.8)
**Clinical stage**	
III<br>IVA	4 (28.5%)<br>10 (71.4%)
**Recurrence**	
Non recurrent<br>Recurrent	8 (57.1%)<br>6 (42.8%)
**Site**	
Carcinoma tongue<br>Carcinoma buccal mucosa<br>Carcinoma oropharynx<br>Carcinoma larynx	3 (21.4%)<br>3 (21.4%)<br>4 (28.5%)<br>4 (28.5%)

The details of each patients including the treatment is given in the next page.

Patient (Sex/Age)	Type and Histology grade	Staging	#mGPS score	Treatment
1 (M/35)	Tongue; PD	T3N1M0 (Stage III)	2	*Platinum re-challenge
2 (M/62)	Tongue; MD	T3N2M0 (Stage IVA)	1	**IC/Surgery/RT
3 (F/47)	Buccal; WD	T3N1M0 (Stage III)	2	*Platinum re-challenge
4 (F/59)	Buccal; MD	T3N2M0 (Stage IVA)	1	IC/Surgery/RT
5 (M/48)	Hypopharynx; MD	T4aN1M0 (Stage IVA)	2	IC/Surgery/RT
6 (M/56)	Buccal; MD	T4aN2M0 (Stage IVA)	2	IC/Surgery/RT
7 (F/54)	Larynx; PD	T3N2M0 (Stage IVA)	2	*Platinum re-challenge
8 (M/65)	Larynx; PD	T4aN1M0 (Stage IVA)	2	*Platinum re-challenge
9 (M/58)	Larynx; MD	T3N1M0 (Stage III)	2	*Platinum re-challenge
10 (M/62)	Hypopharynx; MD	T4bN2M0 (Stage IVA)	2	IC/Surgery/RT
11 (F/48)	Tongue; PD	T3N2M0 (Stage IVA)	1	IC/Surgery/RT
12 (F/63)	Hypopharynx;MD	T4aN1M0 (Stage IVA)	2	IC/Surgery/RT
13 (F/53)	Hypopharynx; PD	T3N2M0 (Stage III)	2	*Platinum re-challenge
14 (M/47)	Larynx; MD	T3N2M0 (Stage IVA)	2	IC/Surgery/RT

The staging has been done based on CT scan Neck/Thorax, and upper gastrointestinal endoscopic/laryngoscopical biopsies.

*These patients were given platinum based chemoradiation followed by surgery. They presented with recurrence. So, they were given cisplatin 75 mg/m2 for 4 cycles. ** These patients opted for induction chemotherapy with cisplatin alone, while waiting for the surgery. Following surgery, radiation was given because of close (< 5 mm) margins, and or extra capsular extension.

#mGPS (modified Glasgow Prognostic Score): Patients who had both high serum CRP (>1mg/dl) and low serum albumin (<3.5 mg/dl) were allocated a mGPS of 2. Patients with one abnormal

value were allocated a GPS of 1. As per the KTC (www.kktel.org) protocol, HNSCC patients with high GPS are encouraged to take cisplatin chemotherapy (3-4 cycles) while they collect funds for the surgery and radiation treatment at urban hospitals.

Abbreviations: WD: well-differentiated; MD: moderately differentiated; PD: poorly differentiated.

Above 14 patients were selected from 32 HNSCC patients registered at KTC, Sualkuchi, Assam based on the selection criteria mentioned in the methodology section. The rest of the 18 subjects were given radiation therapy and or surgery alone, thus we have not included in the study.
