# Supplementary material for: Hypoxia-driven mobilization of altruistic cancer stem cells in platinum-treated head and neck cancer
Source: Front Immunol. 2025 Feb 3;15:1336882. doi: 10.3389/fimmu.2024.1336882 (PMC11830676; doi:10.3389/fimmu.2024.1336882)
Supplement: Supplementary file 3 [file Table2.pdf]

Supplementary Table 2: In vivo limiting dilution assay results of patient-derived EpCAM+/ABCG2+ cells

Patient NO.	EpCAM+ ABCG2+ cell	CSC frequency	EpCAM+ ABCG2- cell	CSC frequency	CD44+/ ALDH+ cells	CSC frequency
2	100 (0/5)	1/1696	1000 (0/5)	1/42398	100 (0/5)	1/987
	500 (1/5)		3000 (0/5)		500 (1/5)	
	1000 (2/5)		5000 (0/5)		1000 (3/5)	
	2000 (4/5)		10,000 (2/5)			
4	100 (1/5)	1/958	1000 (0/5)	1/47688	100 (0/5)	1/1173
	500 (1/5)		3000 (0/6)		500 (1/5)	
	1000 (3/5)		10,000 (2/5)		1000 (3/5)	
	2000 (4/4)				2000 (3/3)	
6	50 (1/5)	1/126	1000 (0/5)	1/31234	100 (1/5)	1/645
	100 (3/5)		3000 (0/6)		500 (2/5)	
	500 (5/5)		10,000 (1/6)		1000 (4/5)	
	1000 (5/5)				2000 (4/4)	
10	50 (1/5)	1/107	1000 (0/10)	1/26214	100 (0/5)	1/856
	100 (3/4)		3000 (1/10)		500 (1/5)	
	500 (5/5)		10,000 (2/5)		1000 (4/5)	
	1000 (5/5)				2000 (5/5)	
12	50 (0/7)	1/129	1000 (0/10)	1/42700	100 (0/5)	1/1057
	100 (4/5)		3000 (1/10)		500 (1/5)	
	500 (5/5)		10,000 (2/10)		1000 (3/5)	
	1000 (5/5)				2000 (5/5)	
14	100 (0/5)	1/896	1000 (0/10)	1/84902	100 (0/5)	1/1649
	500 (2/5)		3000 (0/10)		500 (1/5)	
	1000 (3/5)		10,000 (1/5)		1000 (2/5)	
	2000 (5/5)				2000 (2/2)	

To perform in vivo limiting dilution assay, NOD/SCID mice were injected (subcutaneously) with tumor cells and the animals were observed for 6 months. Moribund mice with visible subcutaneous growth at the site of inoculation were sacrificed; tumors were fixed in formalin and subjected to H&E section to confirm tumor growth. Mice were considered tumor negative if no tumor tissues were identified. CSC frequency was obtained by using extreme limiting dilution assay, ELDA.
