# Supplementary material for: Hypoxia-driven mobilization of altruistic cancer stem cells in platinum-treated head and neck cancer
Source: Front Immunol. 2025 Feb 3;15:1336882. doi: 10.3389/fimmu.2024.1336882 (PMC11830676; doi:10.3389/fimmu.2024.1336882)
Supplement: Supplementary file 4 [file Table3.pdf]

Supplementary Table 3: Serial transplantation assay results of SCC-25 derived EpCAM+/ ABCG2+ cells

Cell source	Cell dose	Tumor/ injection	CSC frequency	Cell source	Cell dose	Tumor/ injection	CSC frequency
EpCAM+/ABCG2- (cisplatin+ group)	1×10^2<br>1×10^3<br>1×10^4<br>1×10^5	0/10<br>0/10<br>1/15<br>2/15	$1/5.1\times10^5$	EpCAM+ ABCG2+ (Secondary Transplant, Cisplatin+)	1×10^2<br>5×10^2<br>1×10^3<br>1×10^4	0/5<br>2/10<br>7/10<br>5/5	1/1278
EpCAM+ABCG2+ (cisplatin+ group)	1×10^2<br>5×10^2<br>1×10^3<br>1×10^4	5/10<br>10/10<br>5/5<br>5/5	1/125	EpCAM+ ABCG2+ (Tertiary transplant, Cisplatin+)	1×10^2<br>5×10^2<br>1×10^3<br>1×10^4	0/5<br>2/10<br>8/10<br>10/10	1/1086
EpCAM+/ABCG2- cells (control)	1×10^2<br>1×10^3<br>1×10^4<br>1×10^5	0/10<br>0/10<br>0/10<br>1/15	$1\times1.1\times10^6$	EpCAM+ABCG2+ (Cisplatin ++)	1×10^2<br>5×10^2<br>1×10^3<br>1×10^4	7/10<br>9/10<br>10/10<br>5/5	1/140
EpCAM+ABCG2+ (control)	1×10^2<br>5×10^2<br>1×10^3<br>1×10^4	0/10<br>4/10<br>6/10<br>5/5	1/1149	EpCAM+/ABCG2- (Cisplatin++)	1×10^2<br>5×10^2<br>1×10^3<br>1×10^4	0/10<br>0/10<br>1/15<br>3/15	$1/3.7\times10^5$
EpCAM+ABCG2+ (Secondary transplant, cisplatin+ + group)	1×10^2<br>5×10^2<br>1×10^3<br>1×10^4	0/5<br>0/5<br>1/5<br>2/5	1/1081	CD44+ALDH1+cells (Cisplatin++)	1×10^2<br>5×10^2<br>1×10^3	1/5<br>4/10<br>4/5	1/769
				CD44+ALDH1+cells (Cisplatin+)	1×10^2<br>5×10^2<br>1×10^3	1/5<br>3/10<br>4/5	1/909
EpCAM+/ABCG2- (Secondary transplant, cisplatin+ + group)	1×10^3<br>1×10^4<br>1×10^5	0/8<br>0/10<br>2/15	$1/7.7\times10^5$	CD44+/ALDH1+ cells (Control)	1×10^2<br>5×10^2<br>1×10^3	0/5<br>1/5<br>3/5	1/1519

To perform serial transplantation assay, the NOD/SCID mice bearing primary subcutaneous tumors were sacrificed to obtain the tumors, which were then dissociated to obtain single tumor cell suspension. These tumor cells were subjected to immunomagnetic or flow cytometry sorting (CD44+/ALDH1+ cells) and the sorted cells were injected subcutaneously to mice to obtain secondary tumors. Then the protocols for the in vivo limiting dilution assay were followed to obtain the CSC frequency of the secondary transplants. The secondary transplant tumors were then subjected to another round of in vivo limiting dilution assay to obtain the tertiary tumor transplant, and their CSC frequency.
